# Antimicrobial Activity of *Arthrospira platensis*-Mediated Gold Nanoparticles against *Streptococcus pneumoniae*: A Metabolomic and Docking Study

**DOI:** 10.3390/ijms251810090

**Published:** 2024-09-19

**Authors:** Lamya Azmy, Ebtesam Al-Olayan, Mohamed A. A. Abdelhamid, Ahmed Zayed, Saly F. Gheda, Khayrya A. Youssif, Hesham A. Abou-Zied, Usama R. Abdelmohsen, Ibraheem B. M. Ibraheem, Seung Pil Pack, Khaled N. M. Elsayed

**Affiliations:** 1 Department of Botany and Microbiology, Faculty of Science, Beni-Suef University, Beni-Suef 62521, Egypt; lamyaazmy@gmail.com (L.A.); ibraheemborie@gmail.com (I.B.M.I.); 2 Department of Zoology, College of Science, King Saud University, Riyadh 11472, Saudi Arabia; eolayan@ksu.edu.sa; 3 Biology Department, Faculty of Education and Arts, Sohar University, Sohar 311, Oman; 4 Department of Biotechnology and Bioinformatics, Korea University, Sejong-Ro 2511, Sejong 30019, Republic of Korea; spack@korea.ac.kr; 5 Department of Pharmacognosy, College of Pharmacy, Tanta University, Elguish Street (Medical Campus), Tanta 31527, Egypt; ahmed.zayed1@pharm.tanta.edu.eg; 6 Department of Botany, Faculty of Science, Tanta University, Tanta 31527, Egypt; sally.gheda@science.tanta.edu.eg; 7 Department of Pharmacognosy, Faculty of Pharmacy, El Saleheya El Gadida University, Sharkia 44813, Egypt; khayrya.youssif@gmail.com; 8 Department of Medicinal Chemistry, Faculty of Pharmacy, Deraya University, New Minia 61111, Egypt; drhesham92@yahoo.com; 9 Department of Pharmacognosy, Faculty of Pharmacy, Deraya University, New Minia 61111, Egypt; usama.ramadan@mu.edu.eg; 10 Department of Pharmacognosy, Faculty of Pharmacy, Minia University, Minia 61519, Egypt

**Keywords:** antimicrobial, gold nanoparticles, *Arthrospira platensis*, *Streptococcus pneumoniae*, protein-protein interaction, molecular docking, metabolics

## Abstract

The emergence of antibiotic-resistant *Streptococcus pneumoniae* necessitates the discovery of novel therapeutic agents. This study investigated the antimicrobial potential of green-synthesized gold nanoparticles (AuNPs) fabricated using *Arthrospira platensis* extract. Characterization using Fourier transform infrared spectroscopy revealed the presence of functional groups such as ketones, aldehydes, and carboxylic acids in the capping agents, suggesting their role in AuNP stabilization. Transmission electron microscopy demonstrated the formation of rod-shaped AuNPs with a mean diameter of 134.8 nm, as determined by dynamic light scattering, and a zeta potential of −27.2 mV, indicating good colloidal stability. The synthesized AuNPs exhibited potent antibacterial activity against *S. pneumoniae*, with a minimum inhibitory concentration (MIC) of 12 μg/mL, surpassing the efficacy of the control antibiotic, tigecycline. To elucidate the underlying mechanisms of action, an untargeted metabolomic analysis of the *A. platensis* extract was performed, identifying 26 potential bioactive compounds belonging to diverse chemical classes. In silico studies focused on molecular docking simulations revealed that compound **22** exhibited a strong binding affinity to *S. pneumoniae* topoisomerase IV, a critical enzyme for bacterial DNA replication. Molecular dynamics simulations further validated the stability of this protein–ligand complex. These findings collectively highlight the promising antimicrobial potential of *A. platensis*-derived AuNPs and their constituent compounds, warranting further investigation for the development of novel anti-pneumococcal therapeutics.

## 1. Introduction

*Streptococcus pneumoniae*, a facultative anaerobic Gram-positive bacteria, is a significant contributor to respiratory diseases. It is responsible for various diseases, including sinusitis, otitis media, *pneumonia*, bacteremia, osteomyelitis, septic arthritis, and meningitis [1]. The highest incidence of *pneumococcal* disease is observed in children younger than two years of age and in adults older than 65 years. It also poses a major threat to patients with heart and spleen issues, HIV, or AIDS as well as to smokers. This underscores the urgent need to address the impact of *S. pneumoniae* on public health [2,3,4]. Antibiotic resistance is widespread worldwide, as the bacteria evolve through complex mechanisms to overcome the drug’s effect, the most prominent of which is the resistance to *pneumococcal* serotypes in children. Moreover, approximately 40% of *pneumococci* generally show multidrug resistance [5,6,7,8,9].

As the World Health Organization has explained, bacteria, fungi, and other resistant microorganisms can resist antimicrobial drugs. Consequently, with the emergence of multidrug resistance (MDR), therefore, most pharmaceutical organizations have focused on developing new and effective antimicrobial agents and treatments to reduce infection and overcome its spread [10,11,12,13,14,15]. Studying the metabolism inside microorganisms is considered an effective measure in identifying vital signs and active compounds within them, and exploiting this technique in pharmaceutical research is a great measure that helps develop pharmaceutical research [16,17].

*Arthrospira platensis* is classified as a prokaryotic blue-green filamentous alga commonly known as spirulina. It is a blue-green alga with high nutritional and medicinal values attributed to the high levels of proteins, essential amino acids, fatty acids, sugars, carotenoids, vitamins, and others. This makes it a fertile field for many biological and industrial uses, in addition to its ability to produce many active substances against cancer, bacteria, and microbes in general. Then, they can be exploited in medical and drug manufacturing [18,19,20,21,22,23]. Moreover, its side effects are considered non-existent compared to other industrial products [24]. In addition, it has succeeded in nanomedicine [25,26] in the form of various types of nanoparticles, including gold nanoparticles (AuNPs), which have proven their effectiveness on many Gram-positive and Gram-negative bacteria [27,28].

AuNPs have received great attention due to their unique properties, as gold is considered an inert and non-toxic metal. Ensuring its safety and eco-friendliness in medical applications Furthermore, its nanoparticles are easily synthesized, making them valuable in various technological and medical fields. They can also be exploited in the field of drug delivery and loading, as they help to accumulate and access disease areas. Furthermore, they are used in biosensing, imaging, tissue engineering [29], and as antibacterial agents given how well they can penetrate bacterial cells during the antibacterial reaction that is affected by the size and shape of the gold nanoparticles [30,31,32,33]. Moreover, synthesizing AuNPs using algae is an excellent solution to all the problems caused by the chemical and physical methods used to create them. Because algae are an easily produced, inexpensive, and environmentally benign autotrophic organism, they also create a variety of physiologically active chemicals that enhance the therapeutic efficacy of nanoparticles. Additionally, the creation of highly specialized nanoparticles is made possible by several types of algae. Algae can absorb a high concentration of heavy metals and reduce them to create nanoparticles thanks to their diverse range of chemicals [34,35,36,37,38,39,40]. Therefore, the unlimited capabilities of algae can be harnessed to biosynthesis nanoparticles. In addition, the general opinion of researchers proved that AuNP–drug conjugates exhibit greater antibacterial activity than individual nanoparticles and drugs [31,41,42,43,44]. However, the *S. pneumoniae* bacteria have yet to be sufficiently studied despite the risk of their resistance to drugs in recent research [45,46].

Moreover, the in silico study via molecular docking provides a simulation of biological systems that enables the manufacture of the drug and knowing a lot of information about it without the hassle of traditional research and subjecting animals to experiments, as they can predict the drug’s purpose and activity and determine its therapeutic targets. It also provides an understanding of its potential mechanisms of action, which supports research and facilitates the drug discovery process [47,48].

Based on what was presented previously, this current study aims to investigate the green synthesis of AuNPs using *A. platensis* methanolic extract and study its effect on *S. pneumoniae*, followed by LC–MS-based metabolic profiling and molecular docking studies to gain insights into the mechanisms underlying the antibacterial effects.

## 2. Results

### 2.1. Characterization of the Synthesized AuNPs

#### 2.1.1. UV-Visible and FTIR Characterization of the Synthesized AuNPs

**Figure 1:**
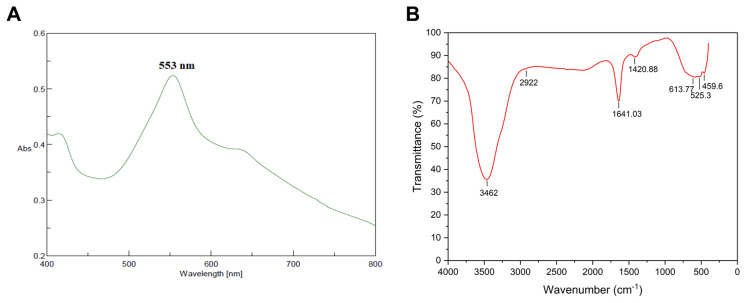
(**A**) UV-Vis spectral analysis of biosynthesized AuNPs using *A. platensis* methanolic extract. (**B**) FTIR spectra of the biosynthesized AuNPs.

The initial formation of AuNPs was visually confirmed by a distinct color change from the reaction mixture to a deep purple hue, a characteristic indicative of surface plasmon resonance (SPR). This visual observation was corroborated by UV-Vis spectroscopy, which revealed a prominent absorption peak centered at 533 nm (Figure 1A). This spectral signature is consistent with previously reported data for AuNPs, providing further evidence of their successful synthesis [49].

Different major peak positions were found in the FTIR spectra at 3462, 2922, 1641, 613, 525, and 459 cm^−1^, as shown in (Figure 1B). The peak at 3462 cm^−1^ was assigned as–OH stretching in alcohols and phenolic compounds with strong hydrogen bonds [50], and 2922 cm^−1^ suggests the presence of stretching vibrations (N-H) of primary and secondary amines [51]. Peaks in the 2840–3000 cm^−1^ range indicate C-H stretching in alkanes [52], while the FTIR peak at 1645 cm^−1^ indicates stretching of the C=C bond. The peak at 613 cm^−1^ is likely to be a stretching of Au–O [50]. The peak at 525 cm^−1^ is assigned to C-C-N stretching in nitriles, and in addition to 459 cm^−1^, it indicates C-H stretching in cycloalkane [53]. 

#### 2.1.2. TEM Characterization of the Synthesized AuNPs

**Figure 2:**
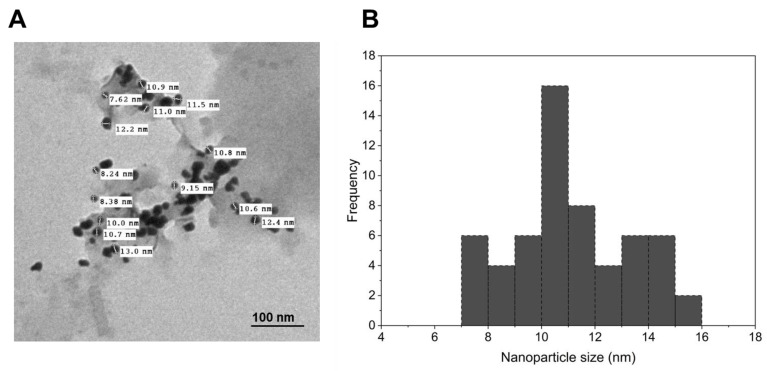
(**A**) TEM analysis showing the shape and size of synthesized AuNPs using *A. platensis* methanolic extract. (**B**) The histogram illustrates the size distribution of the synthesized AuNPs.

TEM plays a crucial role in characterizing the nanoparticles’ size, shape, and morphology. The TEM analysis of AuNPs showed the formation of small rod-shaped nanoparticles in the range of 7–16 nm. Notably, the average size was approximately 10.98 nm. In addition, a histogram of the size distribution is shown in (Figure 2A,B). By providing details about the shape, size range, and average size of the AuNPs, the TEM analysis offers valuable data for comprehending the successful synthesis of nanoparticles.

#### 2.1.3. DLS Technique and Zeta Potential of the Synthesized AuNPs

**Figure 3:**
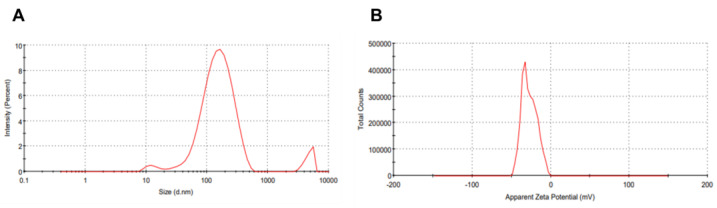
(**A**) Dynamic light scattering analysis of the synthesized AuNPs of *A. platensis* methanolic extract, and (**B**) Zeta potential analysis of the synthesized AuNPs of *A. platensis* methanolic extract.

Understanding the nanoparticles’ surface charges is essential for assessing their stability. Another crucial aspect of nanoparticle characterization is determining their z-average mean [54]. Zeta potential measurements determined that the surface charge of the synthesized AuNPs of the *A. platensis* methanolic extract was negatively charged, with a zeta potential value of −27.2 mV. In addition, the DLS technique determined that the z-average mean diameter of AuNPs of the *A. platensis* methanolic extract was 134.8 nm, with a polydispersity index (PDI) of 0.403 (Figure 3A,B).

#### 2.1.4. Antibacterial Activity

Antibacterial activity was determined by determining the MIC. It is the lowest concentration of AuNPs of the *A. platensis* methanolic extract that can inhibit the visible growth of *S. pneumoniae* after overnight incubation (MIC). The MIC is equal to 12 μg/mL. Detailed MIC values are provided in (Table 1).

**Figure 4:**
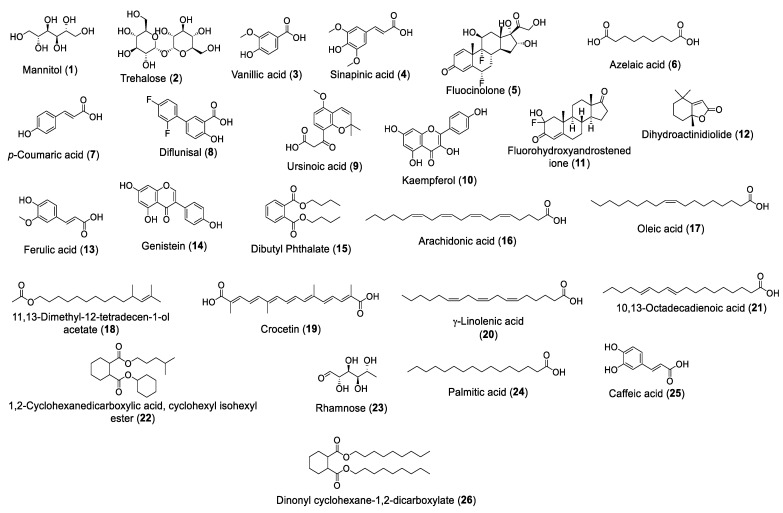
Chemical structures of identified metabolites in *A. platensis* extract as detected in LC–MS total ion chromatogram.

**Figure 5:**
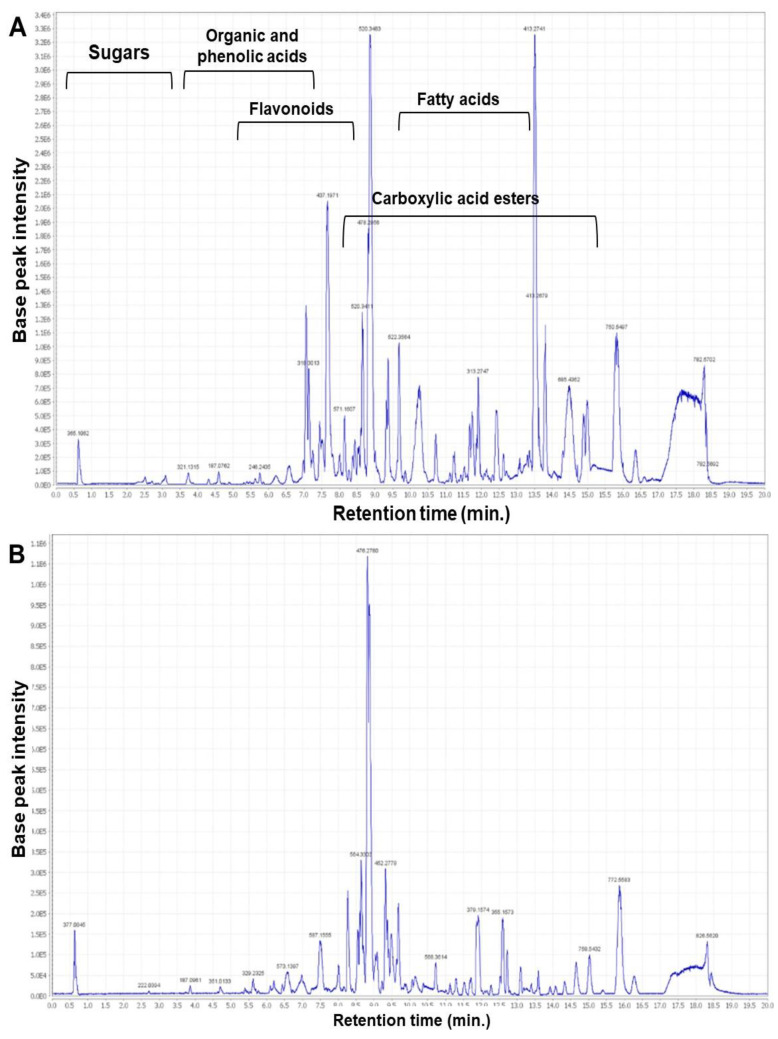
Total ion chromatograms (TIC) of *A. platensis* extract in (**A**) negative and (**B**) positive following analysis by ultra-performance liquid chromatography–tandem mass spectrometry.

The study annotated 26 metabolites based on previous reports and natural product databases. All of these identified metabolites were previously isolated from *A. platensis*. Table 2 lists the 26 metabolites, providing information about their molecular formula, chemical class, molecular weight, peak area, exact mass difference, and the previously reported *A. platensis* source. Additionally, their chemical structures are shown in (Figure 4). Furthermore, total ion chromatograms (TIC) of negative and positive modes are shown in (Figure 5A,B). They belonged mainly to flavonoids, terpenoids, sugars, and fatty acids.

### 2.2. Protein-Protein Interaction Network

#### 2.2.1. Therapeutic Targets for *S. pneumonia* Infections

Based on our experimental findings, we meticulously curated a dataset of 57 target proteins associated with *S. pneumoniae* infections from the NCBI-GEO and PharmGKB databases. These proteins, detailed in Supplementary Table S1, include critical targets and biomarkers central to our investigations into *S. pneumoniae*.

#### 2.2.2. STITCH Database Correlation of *S. pneumoniae* Targets and *A. platensis* Compounds

**Figure 6:**
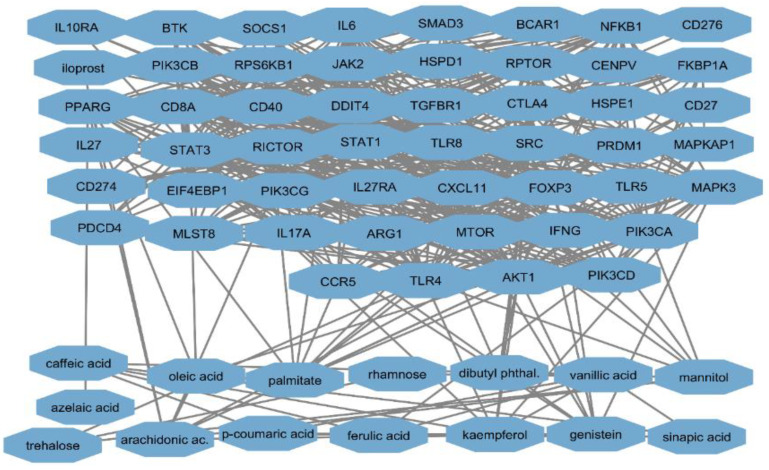
STITCH analysis of *S. pneumoniae* targets and key active compounds in *A. platensis* extract.

In this comprehensive analysis, we utilized the STITCH database to investigate the interactions between key active components in *A. platensis*—specifically, caffeic acid, azelaic acid, trehalose, arachidonic acid, mannitol, oleic acid, rhamnose, ferulic acid, genistein, vanillic acid, and palmitic acid—and protein targets cataloged in GEO and PharmGKB (Figure 6). This initiative aims to advance our understanding of the antibacterial effects of the *A. platensis* extract against *S. pneumoniae* infections. The analysis reveals the synergistic potential of the extract, suggesting a complex interaction between the combined bioactive molecules in the *A. platensis* extract and the proteins related to *S. pneumoniae*.

#### 2.2.3. Protein Network Construction for *S. pneumoniae* Interaction with *A. platensis* Compounds

**Figure 7:**
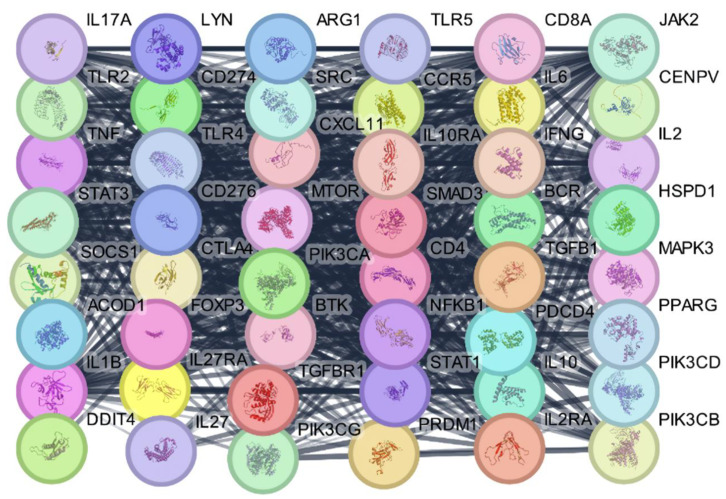
PPI network for *A. platensis* extract against *S. pneumoniae* infections. The nodes represent various proteins involved in biological processes related to the immune response, cell signaling, apoptosis, and metabolic regulation. The color coding of the nodes indicates their functional categories: Red for apoptosis regulators, Green for immune response proteins, Blue for cell signaling proteins, Purple for metabolic enzymes, Yellow for inflammatory response proteins, Orange for transcription regulators, and Pink for proteins involved in cell proliferation and growth.

To develop the protein–protein interaction (PPI) network for this study, we incorporated proteins relevant to *S. pneumoniae* and the active components found in the *A. platensis* extract into the STRING database, version 12.0 (https://string-db.org/cgi/input?sessionId=barlI0uOHF46, accessed on 5 June 2024), to construct preliminary PPI networks elucidating their direct and functional associations. This process facilitated the visualization of the PPI network diagram through Cytoscape software, version 3.10.1. Utilizing the Cytoscape analyzer feature, we established an extensive protein interaction network comprising 48 nodes and 646 interaction linkages, which resulted in an average node connectivity of 26.91. The complexities of this network are detailed in (Figure 7).

#### 2.2.4. Analysis of Overrepresented Gene Ontology Terms

**Figure 8:**
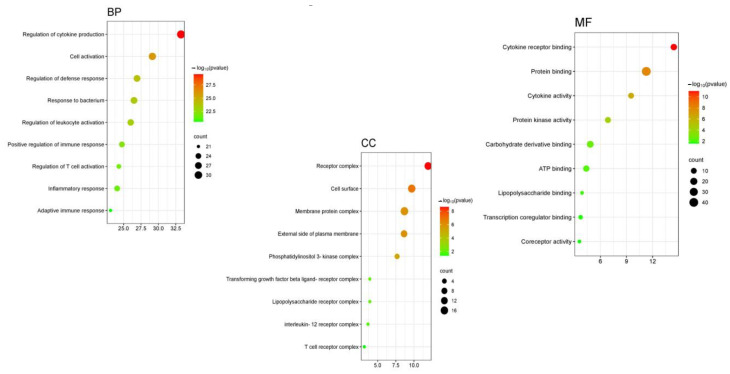
Bubble chart illustrating gene ontology classifications for the antibacterial properties of *A. platensis* extract against *S. pneumoniae*: biological process, cellular component, and molecular function.

The GO enrichment analysis for our study on the antibacterial properties of *A. platensis* extract against *S. pneumoniae* was conducted using ShinyGO v0.80. Our findings demonstrate the significant involvement of proteins in various categories (Figure 8) and Supplementary Table S2. In the biological process (BP) category, key overrepresented GO terms include the regulation of cytokine production, cell activation, regulation of defense response, response to a bacterium, regulation of leukocyte activation, positive regulation of immune response, regulation of T cell activation, inflammatory response, and adaptive immune response. These biological processes are crucial for the defense against *S. pneumoniae* infections, highlighting the involvement of immune responses and specific antibacterial activities. In the cellular component (CC) category, significant terms include receptor complex, cell surface, membrane protein complex, external side of the plasma membrane, phosphatidylinositol 3-kinase complex, transforming growth factor beta ligand-receptor complex, lipopolysaccharide receptor complex, interleukin-12 receptor complex, and T cell receptor complex. These cellular locations are indicative of where *A. platensis* compounds might exert antibacterial effects, particularly in membrane-associated signaling and interaction sites. In the molecular function (MF) category, terms such as cytokine receptor binding, protein binding, cytokine activity, protein kinase activity, carbohydrate derivative binding, ATP binding, lipopolysaccharide binding, transcription coregulator binding, and coreceptor activity were identified. These molecular functions suggest that proteins may engage in pathways that disrupt S. pneumoniae mechanisms by interacting with cytokine and chemokine receptors, essential for immune signaling.

#### 2.2.5. Examination of Predominant KEGG Pathways

**Figure 9:**
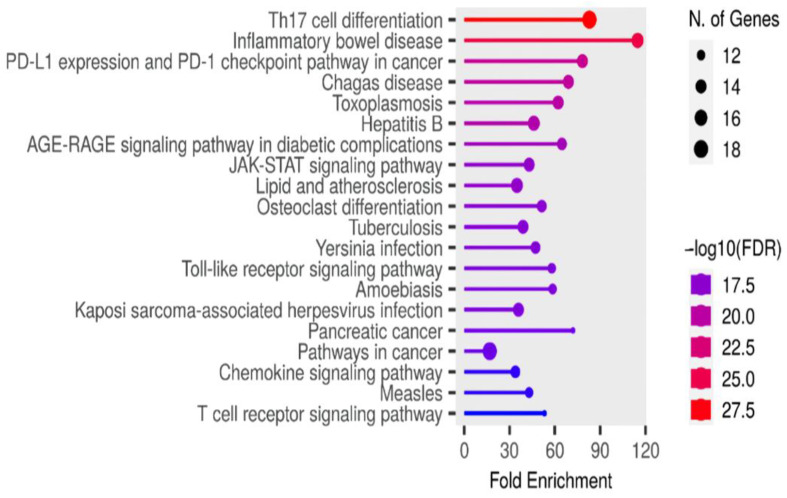
Bar plot illustrating the significant KEGG pathways enriched by *A. platensis* extract, showcasing their potential antibacterial effects against *S. pneumoniae*.

The KEGG pathway analysis is crucial for our study, linking the molecular actions of the *A. platensis* extract to specific biological pathways affected by *S. pneumoniae* infections. Our data, detailed in Supplementary Table S3, are illustrated in a bar plot (Figure 9) and show significant enrichment in pathways such as the Th17 cell differentiation pathway, the toll-like receptor signaling pathway, the JAK-STAT signaling pathway, the Chemokine signaling pathway, and the T cell receptor signaling pathway. These pathways are vital for understanding how the bioactive components in *A. platensis* might influence gene expression and protein interactions to combat *S. pneumoniae* infections.

**Figure 10:**
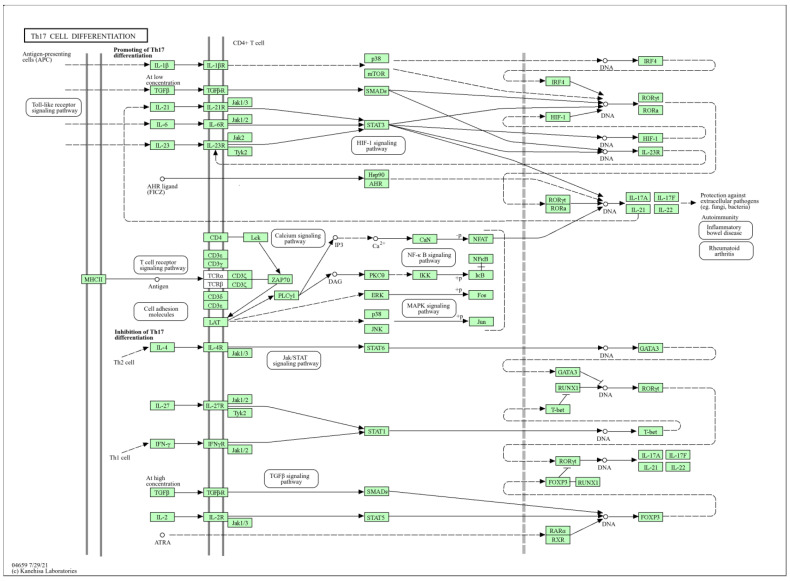
Th17 cell differentiation pathway illustrating how *A. platensis* extract modulates immune responses to combat *S. pneumoniae* infections. Solid lines represent direct interactions or activation steps in the signaling pathways, while dashed lines indicate indirect interactions, regulatory effects, or transcriptional regulation.

Among these, the activation of the Th17 cell differentiation pathway is particularly significant. This pathway is crucial for promoting the differentiation of Th17 cells, which play an essential role in the immune response against extracellular pathogens such as bacteria, including *S. pneumoniae*. Th17 cells are known for their involvement in producing pro-inflammatory cytokines, which help recruit neutrophils and other immune cells to the site of infection, thereby enhancing the antibacterial response. Key elements of the Th17 cell differentiation pathway have been identified (Figure 10), showing how various cytokines and signaling molecules interact to drive the differentiation and activation of Th17 cells. The *A. platensis* extract may enhance this process by modulating the Th17 cell differentiation pathway, potentially increasing the production and activity of Th17 cells. This modulation can lead to an improved immune response against *S. pneumoniae*, promoting the clearance of the infection and preventing the bacteria from evading the immune system.

#### 2.2.6. Identification of Key Hub Genes in the PPI Network

**Figure 11:**
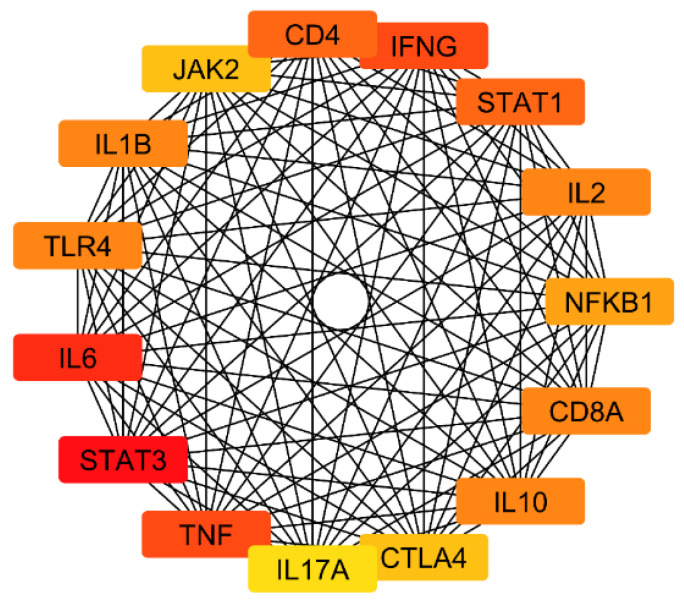
Key hub genes identified in the PPI network related to the antibacterial effects of *A. platensis* extract against *S. pneumoniae*.

Using the CytoHubba plugin, key hub genes within the PPI network related to the antibacterial effects of the *A. platensis* extract against *S. pneumoniae* were identified. These hub genes, including IFNG, IL1B, IL2, IL6, IL10, IL17A, CD4, CD8A, TNF, TLR4, STAT1, STAT3, JAK2, NFKB1, and CTLA4, demonstrate high connectivity and are, therefore, of significant interest (Figure 11).

#### 2.2.7. Molecular Modeling with Topoisomerase IV of *S. pneumoniae*

**Figure 12:**
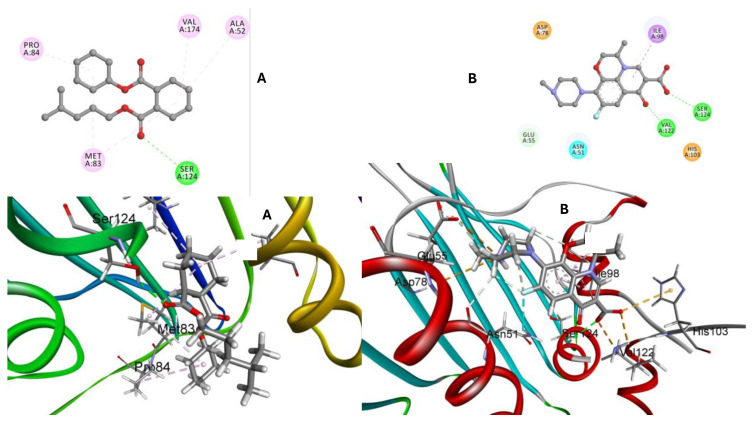
Binding interactions with topoisomerase IV of *S. pneumoniae*. (**A**) interaction map of compound **22** highlighting the conventional hydrogen bonds and alkyl interactions. (**B**) interaction map of Levofloxacin.

Using computational docking, we can simulate the binding of various inhibitors to topoisomerase IV, identifying compounds that inhibit its activity. These inhibitors can be developed into antibacterial drugs, offering a targeted approach to combat *S. pneumoniae* infections. By understanding these interactions, we can develop targeted interventions to inhibit bacterial replication, paving the way for effective treatments against bacterial infections. In this study, key compounds from the *A. platensis* extract (1–26), along with Levofloxacin as a reference, were screened for their ability to bind to the topoisomerase IV of *S. pneumoniae*. The crystal structure of topoisomerase IV was retrieved from the RCSB protein data bank and identified by the entry 4KVB. Molecular docking was performed to evaluate the binding affinity and interactions of these compounds with the enzyme. The docking results are detailed in Supplementary Table S4. From the docking results, compound **22** (1,2-Cyclohexanedicarboxylic acid, cyclohexyl isohexyl ester) emerged as the most promising candidate based on several crucial criteria: strong binding affinity, low RMSD value, and significant interactions with crucial residues. These factors collectively determine its potential as an effective inhibitor. The docking score indicates the binding affinity, where a more negative value reflects a stronger interaction. Compound **22** displayed a notable docking score of −6.84 kcal/mol and an RMSD value of 0.70, indicating a strong interaction and stable binding conformation with topoisomerase IV. The binding interactions of compound **22** with topoisomerase IV were characterized by multiple interactions with crucial active site residues, including MET83, PRO84, SER124, VAL174, and ALA52. These interactions are crucial for binding affinity and are visually detailed in (Figure 12). In comparison, Levofloxacin, a first-line treatment for respiratory *S. pneumoniae* infections, showed a docking score of −5.59 kcal/mol and an RMSD value of 1.943 (Figure 12).

#### 2.2.8. Molecular Dynamics Simulation with Topoisomerase IV of *S. pneumoniae*

**Figure 13:**
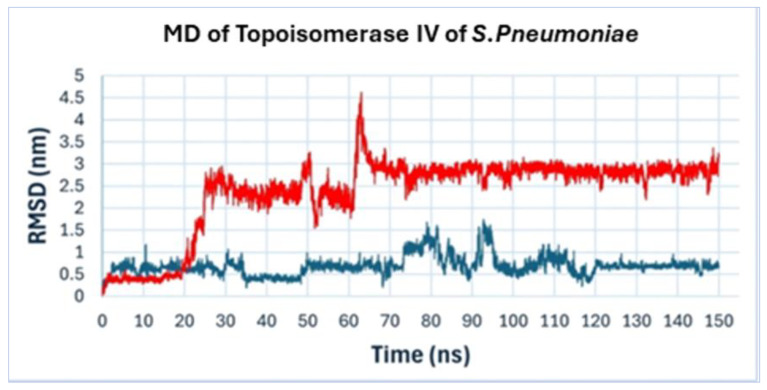
RMSD analysis of topoisomerase IV complexed with compound **22** (blue line) and Levofloxacin (red line) over 150 ns of molecular dynamics simulation.

Molecular dynamics simulations were employed to understand the antibacterial activity of compound **22** from the *A. platensis* extract against *S. pneumoniae*. These simulations assess the stability and efficacy of the binding over time. The molecular dynamics simulations were conducted to evaluate key metrics such as root mean square deviation (RMSD), radius of gyration, and potential energy, thereby assessing the stability and strength of the ligand–receptor interactions. The RMSD analysis was conducted on the molecular dynamics (MD) trajectory of topoisomerase IV complexed with compound **22** and Levofloxacin (as a reference drug). This analysis, after aligning the alpha carbons to the minimized structure, provides insights into the stability of the protein–ligand complexes throughout the simulation. As depicted in (Figure 13), the RMSD values for compound **22** and Levofloxacin fluctuate over the course of 150 ns, demonstrating the dynamic nature of these interactions. Compound **22** showed lower and more stable RMSD values compared to Levofloxacin, indicating a more stable binding conformation.

**Figure 14:**
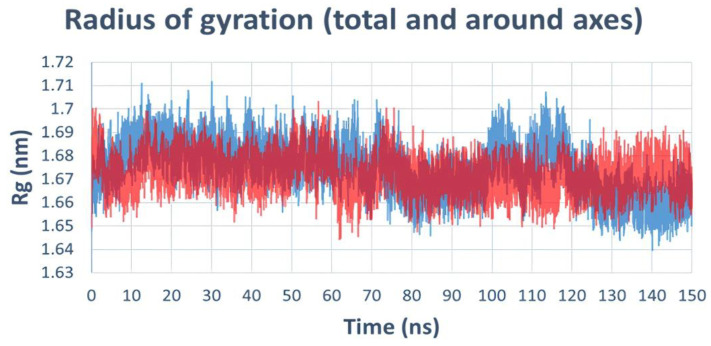
Radius of gyration (Rg) analysis of topoisomerase IV complexed with compound **22** (blue line) and Levofloxacin (red line) over 150 ns of molecular dynamics simulation.

The radius of gyration (Rg) analysis was conducted on the molecular dynamics (MD) trajectory of topoisomerase IV complexed with compound **22** and Levofloxacin (as a reference drug). This analysis provides insights into the compactness and stability of the protein–ligand complexes over the course of 150 ns. As depicted in (Figure 14), the Rg values for both compound **22** and Levofloxacin show fluctuations, indicating the dynamic nature of the protein structures during the simulation. Notably, compound **22** exhibited relatively stable Rg values, suggesting a consistent and compact protein structure compared to Levofloxacin, which displayed higher variability in its Rg values.

**Figure 15:**
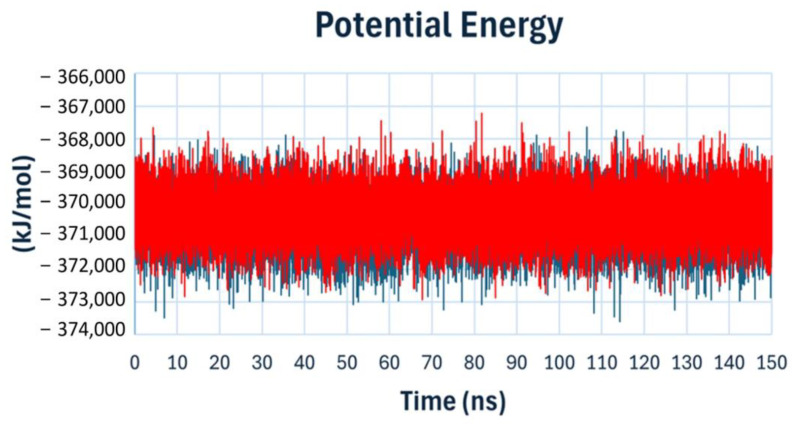
Potential energy analysis of topoisomerase IV complexed with compound **22** (blue line) and Levofloxacin (red line) over 150 ns of molecular dynamics simulation.

The potential energy analysis was conducted on the molecular dynamics (MD) trajectory of topoisomerase IV complexed with compound **22** and Levofloxacin (as a reference drug). This analysis provides further insights into the stability of the protein–ligand complexes over time. As illustrated in (Figure 15), the potential energy values for both compound **22** and Levofloxacin remained relatively stable throughout the 150-nanosecond simulation, suggesting consistent stability of the complexes. Notably, compound **22** exhibited energetically favorable and stable binding conformation.

## 3. Discussion

Based on the findings from the nanoparticle characterization, the formation of AuNPs is excellent and highly stable. The UV-Vis spectrum results showed a spectrum band in the range that confirms the formation of AuNPs similar to those reported in the literature [66,67]. In addition, FTIR is now a crucial tool for understanding functional groups’ role in the interaction between biomolecules and metal particles. It can be used to search for specific biomolecules necessary for stabilizing and capping metal nanoparticles and identify the chemical composition of the AuNPs surface. The observed peaks are mainly attributed to terpenoids, glycosides, flavonoids, tannins, and phenols with functional groups such as ketone, aldehyde, carboxylic acid, and others [68,69], confirming the ability to bind the metal and cap the metal nanoparticles to prevent the agglomeration of the particles [70,71]. Moreover, they are also involved in the bio-reduction of gold ions to nanoparticles and stabilizing AuNPs in the aqueous medium [72]. The presence of these groups increases the stability of the nanoparticles. These metabolites prevent aggregation and pairing of the nanoparticles. These biomolecules are likely responsible for synthesizing nanoparticles’ capping and efficient stabilization. Moreover, Zeta potential analysis was conducted to assess the colloidal stability of the AuNP suspension [54]. This measurement quantifies the electrostatic repulsion between particles, a critical determinant of dispersion stability. The observed negative zeta potential value indicates sufficient electrostatic repulsion to maintain colloidal stability [54,73].

High antimicrobial activity was observed for AuNPs of the *A. platensis* methanolic extract compared with the methanolic extract of *A. platensis* against *S. pneumoniae*. The formulations with lower MICs are more effective in combating bacterial pathogens as antibacterial agents [74]. Determining the MIC value is currently the best tool available to determine the effectiveness of formulations against bacterial strains because bacteria exposed to insufficient concentrations can develop resistance to these formulations. Therefore, determining the MIC value helps prevent the development of drug-resistant microbial strains and improves outcomes for patients [74]. In addition, there are many possibilities that explain the antimicrobial mechanisms of AuNPs, including their ability to penetrate the cell wall, which leads to damage to its components, or the absorption of AuNPs, which depends on their size, as small particles enter the cell, while larger particles work on the surface to dissolve the cells. Moreover, gold particles also change the internal components of microbial cells, which helps eliminate them. In addition, they disturb the balance within the microbial cells, and all of this contributes to causing great harm to them or killing them completely [75,76,77,78]. This supports and confirms the effectiveness of the formulation as a potential drug against *S. pneumoniae*.

Although gold nanoparticles show promise for use in biomedical applications, they may also carry some risks. They can interact with enzymes, disrupt biochemical pathways, and interfere with hormone production, leading to adverse physiological effects [79]. Moreover, at high concentrations or prolonged exposure, gold nanoparticles may be toxic and induce immune system reactions. The long-term effects of these nanoparticles remain to be investigated, and their accumulation in organs and tissues may present unknown health risks over time [80]. Therefore, a thorough evaluation and risk assessment are essential before considering the widespread use of gold nanoparticles in medical applications.

Using LC–MS and metabolic profiling, 26 metabolites were tentatively identified, and by reviewing previous studies, most of the metabolites showed different biological activities. With a focus on activity against bacteria, many metabolites were found to have diverse activity against many types of bacteria, including sinapinic acid (**4**) [81], vanillic acid (**3**) [82,83], *p*-Coumaric acid (**7**) [84,85], kaempferol (**10**) [86], ferulic acid (**13**) [87,88], genistein (**14**) [89], and gamma-linolenic acid (GLA) (**20**) [90]. In addition, diflunisal (**8**) and ursinoic acid (**9**) showed antibacterial activity against *Staphylococcus Aureus* [85,91], arachidonic acid (**16**) affects *Streptococcus mutans* [92], and crocetin (**19**) can be of use in *staphylococcal* infections [93]. On the other hand, azelaic acid’s (**6**) antibacterial activity is pH-dependent [94], and mannitol (**1**) activity is generally considered weak as an antibacterial agent [95].

The results of therapeutic targets for *S. pneumoniae* infections indicated the significance of these proteins in the context of *S. pneumoniae* infections. They highlight their potential as therapeutic targets, supported by experimental evidence from our research on the *A. platensis* extract. Through the results of the STITCH database correlation of *S. pneumoniae* targets and *A. platensis* compounds, this initiative aims to advance our understanding of the antibacterial effects of the *A. platensis* extract against *S. pneumoniae* infections. The analysis reveals the synergistic potential of the extract, suggesting a complex interaction between the combined bioactive molecules in the *A. platensis* extract and the proteins related to *S. pneumoniae*. This interplay is believed to enhance the overall efficacy of the extract over isolated compounds, expanding their therapeutic capabilities against *S. pneumoniae* infections.

In addition, the results of the PPI network highlight the comprehensive analysis of protein interactions pertinent to our study on the antibacterial effects of the *A. platensis* extract against *S. pneumoniae* infections. Furthermore, the GO enrichment analysis result suggests that these molecular functions may engage in pathways that disrupt *S. pneumoniae* mechanisms by interacting with cytokine and chemokine receptors, which are essential for immune signaling.

The KEGG pathway analysis results show that these pathways are vital for understanding how the bioactive components in *A. platensis* might influence gene expression and protein interactions to combat *S. pneumoniae* infections. The significant enrichment in these pathways highlights potential mechanisms of antibacterial action and therapeutic targets for intervention against *S. pneumoniae*.

The results of the identification of key hub genes in the PPI network. These genes in (Figure 11) play crucial roles in the immune response, including interferon production (IFNG), T and B cell activation (IL2, IL17A, CD4, CD8A), immune cell recruitment (IL1B, IL6, IL10), and pro-inflammatory responses (TNF, TLR4). Topoisomerase IV is an essential enzyme in *S. pneumoniae*, crucial for its DNA replication and cell division processes. In the context of an infection, inhibiting this enzyme can significantly impair the ability to proliferate, making it a key target for antibacterial strategies. The *A. platensis* extract contains bioactive compounds that could potentially interfere with the activity of topoisomerase IV, thereby exhibiting antibacterial effects. The hub genes identified in the PPI network may not interact directly with topoisomerase IV, but their modulation can significantly impact the overall immune response, creating a synergistic effect with the direct inhibition of topoisomerase IV. By docking compounds from *A. platensis* with topoisomerase IV, we can explore the potential inhibitory effects on this essential bacterial enzyme. The hypothesis is that bioactive compounds in *A. platensis* can bind to the active sites of topoisomerase IV, inhibiting its activity and thus hindering the DNA replication process of *S. pneumoniae*.

The results of molecular modeling with topoisomerase IV of *S. pneumoniae* have shown that compound **22** has a higher binding affinity, suggesting that it might be a more potent inhibitor of topoisomerase IV compared to Levofloxacin. The strong binding affinity exhibited by compound **22** indicates its potential as a leading candidate for developing new antibacterial agents aimed at combating *S. pneumoniae* infections.

The molecular dynamics simulation with topoisomerase IV of *S. pneumoniae* results showing compound **22** showed lower and more stable RMSD values compared to Levofloxacin, indicating a more stable binding conformation. This stability is crucial for the effective inhibition of topoisomerase IV, suggesting that compound **22** could potentially be more effective in disrupting the bacterial replication process of *S. pneumoniae* compared to Levofloxacin. Additionally, the consistent potential energy values reinforce the findings from the RMSD and Rg analyses, highlighting the stable interaction between compound **22** and topoisomerase IV.

By integrating docking and molecular dynamic studies of *A. platensis* compounds with topoisomerase IV and the modulation of key immune-related hub genes in the PPI network, a comprehensive strategy can be developed to effectively combat *S. pneumoniae* infections. This dual approach enhances both direct antibacterial action and the immune response, offering a robust therapeutic strategy. This dual approach, while theoretically promising, enhances both direct antibacterial action and the immune response, offering a robust therapeutic strategy. We plan to validate this strategy through in vitro and in vivo experiments in our future publications to substantiate its efficacy.

## 4. Materials and Methods

### 4.1. Cyanobacterial Cultures

*A. platensis* (axenic cyanobacterial cultures) was cultured in a Zarrouk medium [96]. A 100 mL inoculum was incubated in 2 L Erlenmeyer flasks containing a sterilized growth medium. Cultures were maintained at 30 ± 2 °C under a photon flux density of 45 μmol m^−2^ s^−1^ and a gaseous environment enriched with 3% CO_2_. Sterile air was supplied through 0.45 μm pore-size filters (Millipore, Burlington, MA, USA). A 12:12 h light:dark photoperiod was employed for 20 days to optimize biomass accumulation. Subsequently, the cyanobacterial biomass was harvested, dried, and weighed for further analysis.

### 4.2. Preparation of Cyanobacterial Extract

To obtain a methanolic extract rich in bioactive compounds from *A. platensis*, 3 g was transferred to a clean flask. Next, 60 mL of 99% methanol (Sigma-Aldrich (Merck KGaA, Darmstadt, Germany)), recognized for efficiently extracting various compounds, was added to the flask. The mixture was stirred for 48 h at 28 ± 2 °C. Finally, the mixture was filtered using a Whatman No. 1 filter paper, and the filtrate collected was the methanolic extract of *A. platensis* used in the next steps [97].

### 4.3. AuNPs Synthesis

AuNPs were synthesized by adding 1 mM of hydrogen tetrachloroaurate (HAuCl_4_) to 10 mL of the gold metal salt, 50 mL of *A. platensis* methanolic extract, and 1 mL of 1N NaOH was added. The reaction was carried out at 28 ± 2 °C. The color change for the extract was observed from a normal colorless solution to a dark purple color. The initial reduction of the gold ions was confirmed by the UV-Vis spectrum of the solution. Then, nanoparticles were characterized by Fourier transform infrared spectroscopy (FTIR) (Bruker, Berlin, Germany), transmission electron microscopy (TEM) (JEOL, Tokyo, Japan), dynamic light scattering technique (DLS), and *Zeta* potential (Malvern Panalytical, Worcestershire, UK).

### 4.4. Characterization of the Synthesized AuNPs

#### 4.4.1. Transmission Electron Microscopy (TEM)

A droplet of the synthesized AuNP solution was dispensed onto carbon-coated copper grids and allowed to air dry under ambient conditions. Transmission electron microscopy (TEM) images were acquired using a JEOL JEM-1010 microscope (Tokyo, Japan) operated at an accelerating voltage of 80 kV [98].

#### 4.4.2. UV-Vis Spectrometry and FTIR

UV-Vis spectroscopy was employed to monitor the formation of AuNPs using a Jasco Tokyo, Japan, V-630 double-beam spectrophotometer within a wavelength range of 400–800 nm. Fourier-transform infrared (FTIR) spectroscopy using a Bruker ALPHA II spectrometer (Berlin, Germany), was utilized to characterize the functional groups bound to the AuNP surface [99].

#### 4.4.3. Dynamic Light Scattering Technique (DLS) Technique and Zeta Potential

The particle size distribution of the nanoparticles was determined using a Malvern Zetasizer Nano ZS (Worcestershire, UK). Measurements were conducted in disposable cuvettes at 25 °C and analyzed with Zeta-sizer 7.01 software [100].

### 4.5. Antibacterial Activity

The antibacterial efficacy was evaluated by determining the minimum inhibitory concentration (MIC), defined as the lowest concentration preventing visible microbial growth after overnight incubation. MIC is a standard method for assessing antimicrobial activity and is clinically relevant for guiding treatment decisions [74,101].

#### 4.5.1. Preparation of Antibiotic Stock Solution and Dilution Range

Ten mg/mL tigecycline stock solution was prepared. Then, a suitable range of concentrations was selected for testing *S. pneumoniae* in the 0.125 to 512 μg/mL range. In that case, the maximum concentration for testing is 512 μg/mL, with serially diluted concentrations using cation-adjusted Mueller–Hinton broth (CAMHB) solution, and the lowest possible dilution concentration is 0.125 μg/mL. In addition, the different tested concentrations were prepared by 10 times the maximum concentration solution by dispensing the appropriate amount of stock solution with a micropipette and diluting it with CAMHB solution [101].

#### 4.5.2. Preparation of Inoculum

A single colony of *S. pneumoniae* obtained from Sigma-Aldrich was dissolved, picked from a Luria-Bertani (LB) LB streak plate in 3 mL LB broth, and incubated overnight at 37 °C, 220 rpm. Then, OD600 (1 OD600 = 10^9^ CFU/mL) was checked with a UV-Vis spectrophotometer (Robonik, Mumbai, India). After that, the microbial solution was diluted with LB broth to get 0.1 OD600 suspension and incubated at 37 °C, 220 rpm, till the mid-log phase (~2 h). Subsequently, 1 mL mid-log phase microbial solution was put in a 1.5 mL Eppendorf tube (Eppendorf, Hamburg, Germany), centrifuged at 6000 rpm for five min, and washed with 1 mL PBS solution. The washing procedure was repeated twice. Afterward, the microbial pellet was dissolved with 1 mL of CAMHB solution. Following, 100 μL the above bacterial solution was mixed with 900 μL PBS, and then OD600 was with a spectrophotometer (Robonik, Mumbai, India). The bacterial concentration can be deduced from the measured value × 10. then, the bacterial concentration was adjusted to 1 × 10^7^ CFU/mL with CAMHB solution (1 OD600 ~10^9^ CFU/mL).

#### 4.5.3. Inoculation and Incubation

Fifty μL of adjusted *S. pneumoniae* strain solution (1 × 10^7^ CFU/mL) was mixed with 850 µL CAMHB and 100 µL of the 10-fold serially diluted tested concentration. In addition, the *E. coli* ATCC25922 bacterial solution was used as a control. Following 900 μL CAMHB and 100 μL solutions of 10-time serial tested concentration were used for OD600 measurement comparison as a negative control. Then, incubated at 37 °C, 220 rpm for 20–24 h. Finally, OD600 was determined with a spectrophotometer. In addition, the MIC endpoint was selected as the lowest concentration in which there is no visible growth of bacteria (no solution turbidity on naked eyes), and the difference between the measured and background OD600 is less than 0.01 [101].

### 4.6. Metabolic Profiling and Peaks Annotation

Metabolomic profiling of the *A. platensis* methanolic extract was conducted following the method by Abdelmohsen et al. [21,102] using an Acquity Ultra Performance Liquid Chromatography system linked to a Synapt G2 HDMS quadrupole time-of-flight hybrid mass spectrometer (UHPLC/MS-MS) (Waters, Milford, MA, USA). Separation of compounds was carried out on a BEH C18 column (2.1 × 100 mm, 1.7 μm particle size; Waters, Milford, MA, USA) with a guard column (2.1 × 5 mm, 1.7 μm particle size) and a linear binary solvent gradient ranging from 0 to 100% eluent B over 6 min at a flow rate of 0.3 mL min^−1^, using 0.1% formic acid in water (*v*/*v*) as solvent A and acetonitrile as solvent B. The injection volume amounted to 2 μL, and the column temperature was maintained at 40 °C. MSConvert software (Version 3) (ProteoWizard, Palo Alto, CA, USA) converted the raw data into separate positive and negative ionization files. Subsequently, the raw data were imported into the data mining software MZmine 2.10 for peak picking, deconvolution, deisotoping, alignment, and formula prediction using the MarinLit database [103].

### 4.7. In Silico Studies

#### 4.7.1. Protein-Protein Interaction Network

To explore the antibacterial potential of the *A. platensis* extract *against S. pneumoniae*, we used data from the Gene Expression Omnibus (GEO) and the Pharmacogenomics Knowledgebase (PharmGKB). These databases provided valuable information on gene expression and genetic variations relevant to antibacterial activities. We employed the STITCH database to investigate the interactions between active compounds in the *A. platensis* extract and protein targets identified from GEO and PharmGKB. This analysis helped identify potential mechanistic pathways through which the extract may exert its antibacterial effects. We constructed a protein–protein interaction (PPI) network using the STRING database [104], focusing on significant functional interactions (combined score > 0.4). The network was visualized and analyzed in Cytoscape [105], emphasizing hub genes critical for antibacterial pathways. These hub genes represent key targets for enhancing antibacterial efficacy against *S. pneumoniae* and could lead to the development of new therapeutic strategies.

#### 4.7.2. Gene Ontology and KEGG Pathway Enrichment

To understand the roles and molecular interactions of genes associated with antibacterial properties against *S. pneumoniae*, we performed gene ontology (GO) and pathway enrichment analyses. These analyses provided insights from three perspectives: biological processes (BP) that describe the various biological functions genes are involved in; cellular components (CC) that identify the specific cellular locations of gene or protein activity; and molecular functions (MF) that define precise molecular activities and interactions. Using ShinyGO, (v0.80.), a web-based tool for GO analysis, with a stringent false discovery rate (FDR) cutoff of less than 0.05, we identified statistically significant gene functions and pathways. The results were visually represented using enrichment bubble plots generated by SRplot, (v1.0.), an online data visualization tool. This approach improved our understanding of gene roles and their interactions in the context of *S. pneumoniae*, highlighting key targets for potential antibacterial interventions. This comprehensive analysis underscores the importance of integrating advanced bioinformatics tools to unravel the complex biological networks associated with *S. pneumoniae*, aiding in the development of effective antibacterial treatments using *A. platensis* extracts.

#### 4.7.3. Docking Studies

To validate the findings from network pharmacology studies, we utilized molecular docking to simulate the binding interactions between proteins and potential antibacterial compounds from the *A. platensis* extract, i.e., the 26 identified metabolites, against *S. pneumoniae*. This process, conducted on the discovery studio client platform [106], identified promising compounds that could effectively interact with key bacterial and host proteins. Protein structures were sourced from the RCSB Protein Data Bank and prepared by removing extraneous molecules and adding necessary modifications like hydrogen atoms and charges. For simplicity, only relevant chains of the protein structures were retained, while other chains were removed using UCSF Chimera [107]. Ligands from the *A. platensis* extract were optimized for interaction studies. Docking simulations identified active sites and assessed binding energies, with the most favorable interactions informing potential antibacterial strategies against *S. pneumoniae*. This method confirmed our network analysis and highlighted effective antibacterial compounds, paving the way for developing novel therapeutic interventions using *A. platensis* extracts.

#### 4.7.4. Molecular Dynamics Simulation

To further validate the docking results, we conducted a molecular dynamics (MD) simulation using GROMACS 2023 [108]. The protein of interest, relevant to interactions with *S. pneumoniae*, was prepared with UCSF Chimera, that was developed by the Resource for Biocomputing, Visualization, and Informatics (RBVI) at the University of California, San Francisco (UCSF), USA. Preparation includes the addition of hydrogen atoms. We used two different force fields: Charmm36 for the protein and CGenFF for the ligands [109]. The entire complex was solvated in a water box with a TIP3P water model, ensuring a minimum extension of 1 nm in all directions. The system was balanced by adding sodium chloride ions for neutralization and supplemented to a final concentration of 150 mM. Initial minimization employed the steepest descent method, followed by 100 ps of NVT (constant number of particles, volume, and temperature) and NPT (constant number of particles, pressure, and temperature) equilibration phases at 300 K and 1.0 bar, respectively, with position restraints on the protein and ligands. Finally, a 150-ns production run was performed with position restraints removed, capturing trajectories every 10 ps for subsequent RMSD (root mean square deviation) and binding energy analyses. This comprehensive setup provided a robust framework for confirming the molecular interactions and stability of the proposed antibacterial compounds from the *A. platensis* extracts against *S. pneumoniae.*

## 5. Conclusions

This research has effectively biosynthesized environmentally friendly AuNPs using *A. platensis*. The biosynthesized AuNPs showed excellent properties and high stability. This supports the biosynthesis of nanoparticles using *A. platensis* and highlights its strong antibacterial activity against *S. pneumoniae*. Notably, the MIC was just 12 μg/mL, suggesting their potential as a novel anti-*S. pneumoniae* agent. The protein–protein interaction (PPI) network revealed crucial hub genes, such as IFNA1, IL7R, CD19, IL2RA, and IFNG, which play significant roles in immune defense mechanisms. Compound **22** from the *A. platensis* extract exhibited a notable docking score of −6.84 kcal/mol and a stable RMSD value of 0.70, indicating strong binding affinity with key residues such as ASN 51, GLU 55, ASP 78, ILE 98, VAL 122, SER 124, and HIS 103. The 150 ns molecular dynamics analysis of RMSD and potential energy indicated a stable ligand–protein complex, with compound **22** showing more stable RMSD values compared to Levofloxacin. This comprehensive analysis highlights the potential of compound **22** as a promising antibacterial agent against *S. pneumoniae*. Hence, the current study sets the stage for developing novel and natural pharmaceutical formulations against *S. pneumoniae*.

## Figures and Tables

**Table 1:** Antimicrobial activity results of methanolic extract of *A. platensis* and AuNPs of *A. platensis* methanolic extract against *S. pneumoniae*.

Samples	Antimicrobial MIC (μg/mL)
AuNPs of *A. platensis* methanolic extract	12
Methanolic extract of *A. platensis*	96
Gold control	N
Ciprofloxacin	6

**Table 2:** List of identified metabolites in *A. platensis* extract by LC–MS in accordance with the previous literature.

No.	Compounds Identified	Molecular Formula	Class	*m*/*z*	RT (min)	M. wt	Mass Error	Source	Ref.
1	Mannitol	C_6_H_14_O_6_	Sugars	181.07047	0.6307197	182.07775	1.25	*A. platensis*	[55]
2	Trehalose	C_12_H_22_O_11_	Sugars	341.10778	0.6315879	342.11505	1.15	*A. platensis*	[56]
3	Vanillic Acid	C_8_H_8_O_4_	Phenolic acids	167.0373	2.930297	168.04458	2.33	*A. platensis*	[57]
4	Sinapinic acid	C_11_H_12_O_5_	Phenolic acids	223.05997	3.415	224.06724	1.23	*A. platensis*	[57]
5	Fluocinolone	C_21_H_26_F_2_O_6_	Pregnadienes	413.17841	3.4952758	412.17113	1.9	*A. platensis*	[58]
6	Azelaic acid	C_9_H_16_O_4_	Dicarboxylic fatty acid	187.0963	3.8552258	188.10358	1.2	*A. platensis.*	[59]
7	*p*-Coumaric acid	C_9_H_8_O_3_	Phenolic Acids	165.05517	3.9338879	164.04789	0.5	*A. platensis*	[60]
8	Diflunisal	C_13_H_8_F_2_O_3_	Salicylates	251.05562	4.5267061	250.04835	4.2	*A. platensis*	[58]
9	Ursinoic acid	C_15_H_16_O_5_	Aromatic oxo acids	275.09124	4.581603	276.09851	1.26	*A. platensis*	[58]
10	Kaempferol	C_15_H_10_O_6_	Flavonoids	285.03948	5.2730576	286.04676	0.9	*A. platensis*	[60]
11	Fluorohydroxyandrostenedione	C_19_H_25_FO_3_	Ketosteroids	319.17085	6.1840621	320.17812	0.65	*A. platensis*	[58]
12	Dihydroactinidiolide	C_11_H_16_O_2_	Terpenoids	181.12295	6.1905045	180.11567	0.6	*A. platensis*	[59]
13	Ferulic acid	C_10_H_10_O_4_	Phenolic acids	195.06605	7.1785288	194.05877	0.8	*A. platensis*	[60]
14	Genistein	C_15_H_10_O_5_	Flavonoids	269.04452	7.7854955	270.05179	1.03	*A. platensis*	[60]
15	Dibutyl Phthalate	C_16_H_22_O_4_	Dicarboxylic acid esters	279.15958	8.7348955	278.1523	1.02	*A. platensis*	[61]
16	Arachidonic acid	C_20_H_32_O_2_	Fatty acids	303.23868	9.4864939	304.24596	5.73	*A. platensis*	[62]
17	Oleic Acid	C_18_H_34_O_2_	Fatty acids	281.24761	9.7342167	282.25489	0.9	*A. platensis*	[56]
18	11,13-Dimethyl-12-tetradecen-1-ol acetate	C_18_H_34_O_2_	Carboxylic acid esters	281.24761	9.7342167	282.25489	0.9	*A. platensis*	[63]
19	Crocetin	C_20_H_24_O_4_	Carotenoids	327.15943	9.8865045	328.1667	0.75	*A. platensis*	[58]
20	Gamma-Linolenic acid (GLA)	C_18_H_30_O_2_	Fatty acids	279.23226	9.8877879	278.2249	0.4	*A. platensis*	[64]
21	10,13-Octadecadienoic acid	C_18_H_32_O_2_	Fatty acids	279.23193	11.925012	280.23921	1.02	*A. platensis*	[65]
22	1,2-Cyclohexanedicarboxylic acid, cyclohexyl isohexyl ester	C_20_H_34_O_4_	Carboxylic acid esters	337.23738	11.929862	338.24466	1.04	*A. platensis*	[63]
23	Rhamnose	C_6_H_12_O_5_	Deoxy Sugars	165.07037	11.954158	164.0631	4.9	*A. platensis*	[55]
24	Palmitic acid	C_16_H_32_O_2_	Fatty acids	255.2319	12.601824	256.23918	1.02	*A. platensis*	[56]
25	Caffeic acid	C_9_H_8_O_4_	Phenolic acids	179.0372	12.631276	180.04448	2.2	*A. platensis*	[60]
26	Dinonyl cyclohexane-1,2-dicarboxylate	C_26_H_48_O_4_	Carboxylic acid esters	423.34688	13.093317	424.35415	1.11	*A. platensis*	[63]

## Data Availability

All data generated or analyzed during this study are included in this published article (and its Supplementary Materials files).

## Supplementary Materials

The following supporting information can be downloaded at: https://www.mdpi.com/article/10.3390/ijms251810090/s1.
