# Supplementary material for: Antimicrobial Activity of Arthrospira platensis-Mediated Gold Nanoparticles against Streptococcus pneumoniae: A Metabolomic and Docking Study
Source: Int J Mol Sci. 2024 Sep 19;25(18):10090. doi: 10.3390/ijms251810090 (PMC11432420; doi:10.3390/ijms251810090)
Supplement: Supplementary file 1 [file ijms-25-10090-s001.zip › ijms-3184008-supplementary.pdf]

Supplementary Data

Table S1. List of Proteins Related to *Streptococcus pneumoniae* Infections.

Protein	Online Library Source
1. IL17A - Interleukin 17A	pharmGKB
2. LYN - Lymphocyte-specific protein tyrosine kinase	pharmGKB
3. ARG1 - Arginase 1	pharmGKB
4. TLR5 - Toll-Like Receptor 5	pharmGKB
5. CD8A - CD8a molecule	pharmGKB
6. JAK2 - Janus Kinase 2	pharmGKB
7. CENPV - Centromere Protein V	pharmGKB
8. IL2 - Interleukin 2	pharmGKB
9. HSPD1 - Heat Shock Protein Family D (Hsp60) Member 1	pharmGKB
10. MAPK3 - Mitogen-Activated Protein Kinase 3	pharmGKB
11. PPARG - Peroxisome Proliferator-Activated Receptor Gamma	pharmGKB
12. PIK3CD - Phosphoinositide-3-Kinase Catalytic Subunit Delta	pharmGKB
13. PIK3CB - Phosphoinositide-3-Kinase Catalytic Subunit Beta	pharmGKB
14. TLR4 - Toll-Like Receptor 4	pharmGKB
15. CD274 - Programmed Death-Ligand 1 (PD-L1)	pharmGKB
16. SRC - SRC Proto-Oncogene, Non-Receptor Tyrosine Kinase	pharmGKB
17. IL6 - Interleukin 6	pharmGKB
18. TNF - Tumor Necrosis Factor	pharmGKB
19. CXCL11 - C-X-C Motif Chemokine Ligand 11	pharmGKB
20. IFNG - Interferon Gamma	pharmGKB
21. CD27 - CD27 molecule	pharmGKB
22. CD276 - CD276 molecule (B7-H3)	pharmGKB
23. MTOR - Mechanistic Target of Rapamycin	pharmGKB
24. SMAD3 - SMAD Family Member 3	pharmGKB
25. STAT3 - Signal Transducer and Activator of Transcription 3	pharmGKB
26. CCR5 - C-C Motif Chemokine Receptor 5	pharmGKB
27. CD4 - CD4 molecule	pharmGKB
28. BCR - B Cell Receptor	pharmGKB
29. SOCS1 - Suppressor of Cytokine Signaling 1	pharmGKB
30. IL10RA - Interleukin 10 Receptor Subunit Alpha	pharmGKB
31. FOXP3 - Forkhead Box P3	pharmGKB
32. TGFBR1 - Transforming Growth Factor Beta Receptor 1	pharmGKB
33. ACOD1 - Aconitate Decarboxylase 1 (also known as IRG1)	NCBI
34. STAT1 - Signal Transducer and Activator of Transcription 1	NCBI
35. NFKB1 - Nuclear Factor Kappa B Subunit 1	NCBI
36. BTK - Bruton's Tyrosine Kinase	NCBI

37. PIK3CA - Phosphoinositide-3-Kinase Catalytic Subunit Alpha	**NCBI**
38. CTLA4 - Cytotoxic T-Lymphocyte Associated Protein 4	**NCBI**
39. PDCD4 - Programmed Cell Death 4	**NCBI**
40. CD40 - CD40 molecule	**NCBI**
41. TGFBR1 - Transforming Growth Factor Beta Receptor 1 (duplicate in list)	**NCBI**
42. IL27RA - Interleukin 27 Receptor Subunit Alpha	**NCBI**
43. PIK3CG - Phosphoinositide-3-Kinase Catalytic Subunit Gamma	**NCBI**
44. PRDM1 - PR Domain Zinc Finger Protein 1 (also known as BLIMP-1)	**NCBI**
45. IL27 - Interleukin 27	**NCBI**
46. DDIT4 - DNA Damage Inducible Transcript 4 (also known as REDD1)	**NCBI**
47. ply - Pneumolysin	**NCBI**
48. pbp2b - Penicillin-Binding Protein 2b	**NCBI**
49. lytA - Autolysin	**NCBI**
50. pspA - Pneumococcal Surface Protein A	**NCBI**
51. nanA - Neuraminidase A	**NCBI**
52. cbpA - Choline-Binding Protein A	**NCBI**
53. pavA - Pneumococcal Adhesin A	**NCBI**
54. spa - Streptococcal Protective Antigen	**NCBI**
55. spn - Streptococcal Phosphoglycerate Kinase	**NCBI**
56. psaA - Pneumococcal Surface Adhesin A	**NCBI**
57. acod1 - Aconitate Decarboxylase 1 (also known as IRG1, duplicate in list)	**NCBI**

PharmGKB (Pharmacogenomics Knowledgebase www.pharmgkb.org).
NCBI (National Center for Biotechnology Information www.ncbi.nlm.nih.gov).

Table S2. GO enrichment entry.

Category	Description
GO Biological Process	Regulation of immune system process
GO Biological Process	Immune system process
GO Biological Process	Regulation of cytokine production
GO Biological Process	Immune response
GO Biological Process	Regulation of immune response
GO Biological Process	Response to another organism
GO Biological Process	Biological process involved in interspecies interaction between organisms
GO Biological Process	Cellular response to organic substance
GO Biological Process	Cell activation
GO Biological Process	Regulation of multicellular organismal process
GO Biological Process	Positive regulation of cytokine production
GO Biological Process	Response to organic substance
GO Biological Process	Response to lipopolysaccharide
GO Biological Process	Positive regulation of gene expression
GO Biological Process	Positive regulation of response to stimulus
GO Biological Process	Regulation of defense response
GO Biological Process	Cellular response to biotic stimulus
GO Biological Process	Cellular response to lipopolysaccharide
GO Biological Process	Response to bacterium
GO Biological Process	Positive regulation of immune system process
GO Biological Process	Defense response
GO Biological Process	Regulation of leukocyte activation
GO Biological Process	Regulation of response to stimulus
GO Biological Process	Response to external stimulus
GO Biological Process	Response to lipid
GO Biological Process	Cell surface receptor signaling pathway
GO Biological Process	Response to oxygen-containing compound
GO Biological Process	Positive regulation of immune response
GO Biological Process	Regulation of lymphocyte activation
GO Biological Process	Regulation of T cell activation
GO Biological Process	Inflammatory response
GO Biological Process	Cellular response to oxygen-containing compound
GO Biological Process	Positive regulation of multicellular organismal process
GO Biological Process	Regulation of response to external stimulus
GO Biological Process	Adaptive immune response
GO Biological Process	Negative regulation of multicellular organismal process
GO Biological Process	Regulation of leukocyte cell-cell adhesion
GO Biological Process	Regulation of inflammatory response
GO Biological Process	Positive regulation of leukocyte activation

GO Biological Process	Response to chemical
GO Biological Process	Signal transduction
GO Biological Process	Regulation of cell population proliferation
GO Biological Process	Immune system development
GO Biological Process	Positive regulation of lymphocyte activation
GO Biological Process	Defense response to other organism
GO Biological Process	Positive regulation of biological process
GO Biological Process	Leukocyte activation
GO Biological Process	Cellular response to lipid
GO Biological Process	Response to cytokine
GO Biological Process	Response to stress
GO Biological Process	Cellular response to cytokine stimulus
GO Biological Process	Regulation of response to stress
GO Biological Process	Positive regulation of cell-cell adhesion
GO Biological Process	Regulation of leukocyte proliferation
GO Biological Process	Positive regulation of leukocyte cell-cell adhesion
GO Biological Process	Regulation of cell adhesion
GO Biological Process	Cellular response to stimulus
GO Biological Process	Regulation of lymphocyte proliferation
GO Biological Process	Positive regulation of T cell activation
GO Biological Process	Positive regulation of cell adhesion
GO Biological Process	Regulation of hemopoiesis
GO Biological Process	Hematopoietic or lymphoid organ development
GO Biological Process	Negative regulation of cell population proliferation
GO Biological Process	Positive regulation of cell population proliferation
GO Biological Process	Regulation of adaptive immune response based on somatic recombination of immune receptors built from immunoglobulin superfamily domains
GO Biological Process	Regulation of developmental process
GO Biological Process	Positive regulation of defense response
GO Biological Process	Regulation of multicellular organismal development
GO Biological Process	Cytokine-mediated signaling pathway
GO Biological Process	Positive regulation of response to external stimulus
GO Biological Process	Regulation of cell differentiation
GO Biological Process	Positive regulation of cell differentiation
GO Biological Process	Positive regulation of cellular process
GO Biological Process	Regulation of leukocyte differentiation
GO Biological Process	Leukocyte differentiation
GO Biological Process	Regulation of cell death
GO Biological Process	Response to stimulus
GO Biological Process	Negative regulation of immune system process
GO Biological Process	Response to organic cyclic compound
GO Biological Process	Hemopoiesis

GO Biological Process	Regulation of immune effector process
GO Biological Process	Regulation of apoptotic process
GO Biological Process	Regulation of T cell proliferation
GO Biological Process	Regulation of gene expression
GO Biological Process	Positive regulation of developmental process
GO Biological Process	Regulation of interleukin-6 production
GO Biological Process	Positive regulation of adaptive immune response based on somatic recombination of immune receptors built from immunoglobulin superfamily domains
GO Biological Process	Positive regulation of macromolecule metabolic process
GO Biological Process	Positive regulation of metabolic process
GO Biological Process	System development
GO Biological Process	Positive regulation of cell migration
GO Biological Process	Regulation of interferon-gamma production
GO Biological Process	Positive regulation of leukocyte proliferation
GO Biological Process	Negative regulation of response to stimulus
GO Biological Process	Lymphocyte activation
GO Biological Process	Apoptotic process
GO Biological Process	Regulation of leukocyte mediated immunity
GO Biological Process	Response to endogenous stimulus
GO Biological Process	Regulation of cell migration
GO Biological Process	Regulation of signal transduction
GO Biological Process	Negative regulation of metabolic process
GO Biological Process	Leukocyte activation involved in immune response
GO Biological Process	Positive regulation of cell death
GO Biological Process	Negative regulation of cellular process
GO Biological Process	Regulation of smooth muscle cell proliferation
GO Biological Process	Response to organonitrogen compound
GO Biological Process	Positive regulation of immune effector process
GO Biological Process	Regulation of metabolic process
GO Biological Process	Regulation of interleukin-17 production
GO Biological Process	Negative regulation of cytokine production
GO Biological Process	Regulation of response to biotic stimulus
GO Biological Process	Negative regulation of biological process
GO Biological Process	Negative regulation of gene expression
GO Biological Process	Immune effector process
GO Biological Process	Positive regulation of signal transduction
GO Biological Process	Regulation of tumor necrosis factor superfamily cytokine production
GO Biological Process	T cell activation
GO Biological Process	Negative regulation of developmental process
GO Biological Process	Regulation of macromolecule metabolic process
GO Biological Process	Immune response-regulating signaling pathway
GO Biological Process	Regulation of phosphate metabolic process

GO Biological Process	Regulation of phosphorylation
GO Biological Process	Positive regulation of molecular function
GO Biological Process	Cellular response to endogenous stimulus
GO Biological Process	Regulation of protein phosphorylation
GO Biological Process	Negative regulation of immune response
GO Biological Process	Negative regulation of lymphocyte proliferation
GO Biological Process	Regulation of production of molecular mediator of immune response
GO Biological Process	Positive regulation of apoptotic process
GO Biological Process	Negative regulation of leukocyte activation
GO Biological Process	Positive regulation of interleukin-6 production
GO Biological Process	Innate immune response
GO Biological Process	Positive regulation of inflammatory response
GO Biological Process	Positive regulation of lymphocyte proliferation
GO Biological Process	Negative regulation of inflammatory response
GO Biological Process	Regulation of DNA-binding transcription factor activity
GO Biological Process	Response to peptide
GO Biological Process	Myeloid leukocyte activation
GO Biological Process	Intracellular signal transduction
GO Biological Process	Animal organ development
GO Biological Process	Anatomical structure development
GO Biological Process	Negative regulation of cell differentiation
GO Biological Process	Regulation of molecular function
GO Biological Process	Cellular response to organonitrogen compound
GO Biological Process	Regulation of tumor necrosis factor production
GO Biological Process	Negative regulation of lymphocyte activation
GO Biological Process	Negative regulation of defense response
GO Biological Process	Cell population proliferation
GO Biological Process	Positive regulation of interferon-gamma production
GO Biological Process	Regulation of lymphocyte mediated immunity
GO Biological Process	Regulation of cellular process
GO Biological Process	Mononuclear cell differentiation
GO Biological Process	Positive regulation of leukocyte differentiation
GO Biological Process	Leukocyte migration
GO Biological Process	Positive regulation of protein phosphorylation
GO Biological Process	Positive regulation of smooth muscle cell proliferation
GO Biological Process	Negative regulation of macromolecule metabolic process
GO Biological Process	Response to hormone
GO Biological Process	Positive regulation of catalytic activity
GO Biological Process	Enzyme-linked receptor protein signaling pathway
GO Biological Process	Cell migration
GO Biological Process	Regulation of kinase activity
GO Biological Process	Positive regulation of phosphate metabolic process
GO Biological Process	Regulation of transferase activity

GO Biological Process	Positive regulation of DNA-binding transcription factor activity
GO Biological Process	Regulation of protein modification process
GO Biological Process	Regulation of B cell activation
GO Biological Process	Regulation of protein kinase activity
GO Biological Process	Response to peptide hormone
GO Biological Process	Response to virus
GO Biological Process	Regulation of protein metabolic process
GO Biological Process	Regulation of lymphocyte differentiation
GO Biological Process	Regulation of nitric oxide biosynthetic process
GO Biological Process	Regulation of interleukin-10 production
GO Biological Process	Response to abiotic stimulus
GO Biological Process	Positive regulation of interleukin-1 beta production
GO Biological Process	Response to mechanical stimulus
GO Biological Process	Phosphorylation
GO Biological Process	Cellular response to growth factor stimulus
GO Biological Process	Negative regulation of cellular metabolic process
GO Biological Process	Negative regulation of response to external stimulus
GO Biological Process	Positive regulation of gliogenesis
GO Biological Process	Cellular response to peptide
GO Biological Process	Positive regulation of T cell proliferation
GO Biological Process	Positive regulation of protein metabolic process
GO Biological Process	Positive regulation of protein modification process
GO Biological Process	Regulation of interleukin-1 production
GO Biological Process	Cellular response to organic cyclic compound
GO Biological Process	Regulation of alpha-beta T cell activation
GO Biological Process	Lipopolysaccharide-mediated signaling pathway
GO Biological Process	Regulation of intracellular signal transduction
GO Biological Process	Negative regulation of apoptotic process
GO Biological Process	Regulation of cytokine production involved in immune response
GO Biological Process	Positive regulation of cellular metabolic process
GO Biological Process	Regulation of immunoglobulin production
GO Biological Process	Regulation of biological quality
GO Biological Process	Positive regulation of nitric oxide biosynthetic process
GO Biological Process	Regulation of MAP kinase activity
GO Biological Process	Regulation of peptidyl-tyrosine phosphorylation
GO Biological Process	Glial cell differentiation
GO Biological Process	Regulation of MAPK cascade
GO Biological Process	Negative regulation of T cell activation
GO Biological Process	Myeloid cell differentiation
GO Biological Process	Negative regulation of cell-cell adhesion
GO Biological Process	Regulation of cellular metabolic process
GO Biological Process	Positive regulation of peptidyl-tyrosine phosphorylation
GO Biological Process	Myeloid leukocyte differentiation

GO Biological Process	Positive regulation of cellular biosynthetic process
GO Biological Process	Regulation of receptor signaling pathway via STAT
GO Biological Process	Regulation of interleukin-8 production
GO Biological Process	Cell differentiation
GO Biological Process	Multicellular organismal process
GO Biological Process	Regulation of nitrogen compound metabolic process
GO Biological Process	Positive regulation of leukocyte mediated immunity
GO Biological Process	Lymphocyte differentiation
GO Biological Process	Positive regulation of transferase activity
GO Biological Process	Response to ketone
GO Biological Process	Response to steroid hormone
GO Biological Process	Positive regulation of intracellular signal transduction
GO Biological Process	Negative regulation of cell adhesion
GO Biological Process	Leukocyte proliferation
GO Biological Process	Positive regulation of transcription, DNA-templated
GO Biological Process	Positive regulation of miRNA metabolic process
GO Biological Process	Regulation of myeloid cell differentiation
GO Biological Process	Regulation of chemokine production
GO Biological Process	Protein phosphorylation
GO Biological Process	Positive regulation of kinase activity
GO Biological Process	Positive regulation of macromolecule biosynthetic process
GO Biological Process	Regulation of catalytic activity
GO Biological Process	Positive regulation of tumor necrosis factor production
GO Biological Process	Regulation of transport
GO Biological Process	Regulation of phagocytosis
GO Biological Process	Positive regulation of nitrogen compound metabolic process
GO Biological Process	Toll-like receptor signaling pathway
GO Biological Process	Gland development
GO Biological Process	Regulation of protein kinase B signaling
GO Biological Process	Response to corticosteroid
GO Biological Process	Regulation of immunoglobulin mediated immune response
GO Biological Process	Regulation of interleukin-12 production
GO Biological Process	Cellular response to peptide hormone stimulus
GO Biological Process	Negative regulation of phosphate metabolic process
GO Biological Process	Adaptive immune response based on somatic recombination of immune receptors built from immunoglobulin superfamily domains
GO Biological Process	Positive regulation of interleukin-8 production
GO Biological Process	T cell differentiation
GO Biological Process	Positive regulation of protein kinase B signaling
GO Biological Process	Positive regulation of nucleobase-containing compound metabolic process
GO Biological Process	Regulation of regulatory T cell differentiation
GO Biological Process	Positive regulation of lymphocyte mediated immunity

GO Biological Process	Regulation of T cell differentiation
GO Biological Process	Response to wounding
GO Biological Process	Positive regulation of chemokine production
GO Biological Process	Regulation of localization
GO Biological Process	Regulation of myeloid leukocyte differentiation
GO Biological Process	Lymphocyte proliferation
GO Biological Process	Negative regulation of interleukin-17 production
GO Biological Process	Negative regulation of T cell proliferation
GO Biological Process	Activation of immune response
GO Biological Process	Regulation of tyrosine phosphorylation of STAT protein
GO Biological Process	Regulation of CD4-positive, alpha-beta T cell activation
GO Biological Process	Regulation of epithelial cell proliferation
GO Biological Process	Positive regulation of interleukin-10 production
GO Biological Process	Positive regulation of lymphocyte differentiation
GO Biological Process	Negative regulation of phosphorylation
GO Biological Process	Immune response-activating cell surface receptor signaling pathway
GO Biological Process	Negative regulation of protein metabolic process
GO Biological Process	Positive regulation of production of molecular mediator of immune response
GO Biological Process	Cellular response to hormone stimulus
GO Biological Process	Regulation of endothelial cell proliferation
GO Biological Process	Regulation of protein serine/threonine kinase activity
GO Biological Process	Positive regulation of nitric-oxide synthase biosynthetic process
GO Biological Process	Homeostatic process
GO Biological Process	Regulation of activated T cell proliferation
GO Biological Process	Regulation of nucleobase-containing compound metabolic process
GO Biological Process	Antigen receptor-mediated signaling pathway
GO Biological Process	Leukocyte mediated immunity
GO Biological Process	Negative regulation of protein modification process
GO Biological Process	Response to glucocorticoid
GO Biological Process	Positive regulation of transcription by RNA polymerase II
GO Biological Process	Negative regulation of signal transduction
GO Biological Process	Positive regulation of protein transport
GO Biological Process	Negative regulation of miRNA-mediated gene silencing
GO Biological Process	Positive regulation of miRNA transcription
GO Biological Process	Defense response to bacterium
GO Biological Process	Regulation of acute inflammatory response
GO Biological Process	Positive regulation of receptor signaling pathway via STAT
GO Biological Process	MyD88-dependent toll-like receptor signaling pathway
GO Biological Process	Positive regulation of protein kinase activity
GO Biological Process	Positive regulation of neurogenesis
GO Biological Process	Receptor signaling pathway via JAK-STAT
GO Biological Process	Positive regulation of epithelial to mesenchymal transition

GO Biological Process	Regulation of leukocyte apoptotic process
GO Biological Process	Regulation of epithelial cell migration
GO Biological Process	Positive regulation of lipid metabolic process
GO Biological Process	Transmembrane receptor protein tyrosine kinase signaling pathway
GO Biological Process	Regulation of calcidiol 1-monooxygenase activity
GO Biological Process	Positive regulation of regulatory T cell differentiation
GO Biological Process	Negative regulation of protein phosphorylation
GO Biological Process	Regulation of primary metabolic process
GO Biological Process	Positive regulation of myeloid cell differentiation
GO Biological Process	Regulation of vesicle-mediated transport
GO Biological Process	Negative regulation of catabolic process
GO Biological Process	Positive regulation of cytokine production involved in inflammatory response
GO Biological Process	Regulation of cytokine production involved in inflammatory response
GO Biological Process	Cellular response to ketone
GO Biological Process	Cellular response to interferon-gamma
GO Biological Process	Myeloid leukocyte mediated immunity
GO Biological Process	Regulation of protein secretion
GO Biological Process	Regulation of lipid metabolic process
GO Biological Process	Regulation of transcription, DNA-templated
GO Biological Process	Cellular response to stress
GO Biological Process	Positive regulation of acute inflammatory response
GO Biological Process	Myeloid cell activation involved in immune response
GO Biological Process	Peptidyl-tyrosine phosphorylation
GO Biological Process	Astrocyte differentiation
GO Biological Process	Regulation of vascular associated smooth muscle cell proliferation
GO Biological Process	Extrinsic apoptotic signaling pathway
GO Biological Process	Positive regulation of interleukin-17 production
GO Biological Process	Glial cell development
GO Biological Process	Positive regulation of cellular component organization
GO Biological Process	Positive regulation of dephosphorylation
GO Biological Process	Cellular response to interleukin-6
GO Biological Process	Positive regulation of tyrosine phosphorylation of STAT protein
GO Biological Process	Positive regulation of MAPK cascade
GO Biological Process	Positive regulation of response to biotic stimulus
GO Biological Process	Cellular response to chemical stress
GO Biological Process	Regulation of B cell proliferation
GO Biological Process	Positive regulation of proteolysis
GO Biological Process	Positive regulation of leukocyte apoptotic process
GO Biological Process	Negative regulation of immune effector process
GO Biological Process	Regulation of interleukin-23 production
GO Biological Process	Regulation of alpha-beta T cell differentiation
GO Biological Process	Phosphate-containing compound metabolic process

GO Biological Process	Regulation of protein transport
GO Biological Process	Regulation of angiogenesis
GO Biological Process	Liver development
GO Biological Process	Response to insulin
GO Biological Process	Regulation of cellular component organization
GO Biological Process	Negative regulation of cytokine production involved in immune response
GO Biological Process	Regulation of tissue remodeling
GO Biological Process	Positive regulation of phagocytosis
GO Biological Process	Stress-activated MAPK cascade
GO Biological Process	Cellular response to mechanical stimulus
GO Biological Process	Transmembrane receptor protein serine/threonine kinase signaling pathway
GO Biological Process	Regulation of dephosphorylation
GO Biological Process	Positive regulation of epithelial cell proliferation
GO Biological Process	Negative regulation of B cell activation
GO Biological Process	Cell development
GO Biological Process	Regulation of receptor signaling pathway via JAK-STAT
GO Biological Process	Negative regulation of molecular function
GO Biological Process	Glial cell activation
GO Biological Process	Myeloid leukocyte migration
GO Biological Process	Regulation of isotype switching
GO Biological Process	Regulation of catabolic process
GO Biological Process	Regulation of DNA metabolic process
GO Biological Process	MAPK cascade
GO Biological Process	Leukocyte degranulation
GO Biological Process	Animal organ morphogenesis
GO Biological Process	Cellular response to external stimulus
GO Biological Process	Apoptotic signaling pathway
GO Biological Process	interleukin-6-mediated signaling pathway
GO Biological Process	Leukocyte chemotaxis
GO Biological Process	Regulation of inflammatory response to antigenic stimulus
GO Biological Process	Cellular response to virus
GO Biological Process	Positive regulation of epithelial cell migration
GO Biological Process	Regulation of secretion by cell
GO Biological Process	Regulation of cellular biosynthetic process
GO Biological Process	Positive regulation of interleukin-12 production
GO Biological Process	T cell selection
GO Biological Process	Regulation of small molecule metabolic process
GO Biological Process	Central nervous system development
GO Biological Process	CD4-positive, alpha-beta T cell lineage commitment
GO Biological Process	Positive regulation of MHC class II biosynthetic process
GO Biological Process	Leukocyte homeostasis

GO Biological Process	Response to dexamethasone
GO Biological Process	Wound healing
GO Biological Process	Positive regulation of B cell activation
GO Biological Process	Positive regulation of NF-kappaB transcription factor activity
GO Biological Process	Positive regulation of receptor signaling pathway via JAK-STAT
GO Biological Process	Negative regulation of miRNA maturation
GO Biological Process	Negative regulation of interleukin-6 production
GO Biological Process	Regulation of alpha-beta T cell proliferation
GO Biological Process	Positive regulation of transport
GO Biological Process	Cytokine production
GO Biological Process	Homeostasis of number of cells
GO Biological Process	Positive regulation of glial cell proliferation
GO Biological Process	Regulation of type I interferon production
GO Biological Process	T cell receptor signaling pathway
GO Biological Process	Neurogenesis
GO Biological Process	Positive regulation of endopeptidase activity
GO Biological Process	Regulation of monooxygenase activity
GO Biological Process	Gland morphogenesis
GO Biological Process	Negative regulation of B cell proliferation
GO Biological Process	Positive regulation of SMAD protein signal transduction
GO Biological Process	Positive regulation of calcidiol 1-monooxygenase activity
GO Biological Process	Positive regulation of immunoglobulin production
GO Biological Process	Negative regulation of nitrogen compound metabolic process
GO Biological Process	Negative regulation of leukocyte differentiation
GO Biological Process	Regulation of CD4-positive, alpha-beta T cell differentiation
GO Biological Process	Regulation of binding
GO Biological Process	Tolerance induction
GO Biological Process	Positive regulation of cellular component biogenesis
GO Biological Process	Regulation of cellular catabolic process
GO Biological Process	Response to hypoxia
GO Biological Process	JNK cascade
GO Biological Process	Positive regulation of myeloid leukocyte differentiation
GO Biological Process	Negative regulation of MAP kinase activity
GO Biological Process	Regulation of B cell apoptotic process
GO Biological Process	Positive regulation of humoral immune response
GO Biological Process	Regulation of carbohydrate metabolic process
GO Biological Process	B cell activation
GO Biological Process	Negative regulation of intracellular signal transduction
GO Biological Process	Anatomical structure morphogenesis
GO Biological Process	Positive regulation of MAP kinase activity
GO Biological Process	Positive regulation of T cell differentiation
GO Biological Process	Response to amino acid
GO Biological Process	Negative regulation of heart contraction

GO Biological Process	Blood circulation
GO Biological Process	Macrophage activation
GO Biological Process	Regulation of CD4-positive, alpha-beta T cell proliferation
GO Biological Process	Negative regulation of tumor necrosis factor superfamily cytokine production
GO Biological Process	Positive regulation of nucleocytoplasmic transport
GO Biological Process	Regulation of lymphocyte apoptotic process
GO Biological Process	Regulation of ERK1 and ERK2 cascade
GO Biological Process	Mast cell degranulation
GO Biological Process	Regulation of membrane protein ectodomain proteolysis
GO Biological Process	Response to interleukin-1
GO Biological Process	Phosphatidylinositol phosphate biosynthetic process
GO Biological Process	Response to xenobiotic stimulus
GO Biological Process	Animal organ regeneration
GO Biological Process	Positive regulation of NIK/NF-kappaB signaling
GO Biological Process	I-kappaB kinase/NF-kappaB signaling
GO Biological Process	Lymphocyte activation involved in immune response
GO Biological Process	Defense response to Gram-positive bacterium
GO Biological Process	Regulation of generation of precursor metabolites and energy
GO Biological Process	Tube development
GO Biological Process	Regulation of osteoclast differentiation
GO Biological Process	Cellular response to dexamethasone stimulus
GO Biological Process	Tolerance induction to self antigen
GO Biological Process	T cell proliferation
GO Biological Process	Macrophage differentiation
GO Biological Process	Cellular process
GO Biological Process	Regulation of neuron death
GO Biological Process	Cellular response to abiotic stimulus
GO Biological Process	Peptidyl-amino acid modification
GO Biological Process	Regulation of glial cell differentiation
GO Biological Process	Liver regeneration
GO Biological Process	Negative regulation of cell junction assembly
GO Biological Process	Regulation of T-helper 17 type immune response
GO Biological Process	Regulation of proteolysis
GO Biological Process	Positive regulation of hydrolase activity
GO Biological Process	Positive regulation of protein serine/threonine kinase activity
GO Biological Process	Microglial cell activation
GO Biological Process	Regulation of interferon-alpha production
GO Biological Process	Regulation of peptidyl-serine phosphorylation
GO Biological Process	Vascular endothelial growth factor production
GO Biological Process	Macrophage derived foam cell differentiation
GO Biological Process	Regulation of vitamin D biosynthetic process
GO Biological Process	Regulation of leukocyte migration

GO Biological Process	Regulation of anatomical structure morphogenesis
GO Biological Process	Tissue development
GO Biological Process	Nervous system development
GO Biological Process	Positive regulation of small molecule metabolic process
GO Biological Process	Positive regulation of monooxygenase activity
GO Biological Process	Regulation of innate immune response
GO Biological Process	Regulation of autophagy
GO Biological Process	Cellular response to transforming growth factor beta stimulus
GO Biological Process	Cellular response to steroid hormone stimulus
GO Biological Process	Regeneration
GO Biological Process	Activation of cysteine-type endopeptidase activity involved in apoptotic process
GO Biological Process	Regulation of phosphatase activity
GO Biological Process	Positive regulation of phosphatase activity
GO Biological Process	Astrocyte development
GO Biological Process	Regulation of cellular localization
GO Biological Process	Positive regulation of vascular associated smooth muscle cell proliferation
GO Biological Process	Regulation of T cell mediated immunity
GO Biological Process	Cell fate commitment
GO Biological Process	Response to extracellular stimulus
GO Biological Process	Negative regulation of autophagy
GO Biological Process	Regulation of cellular component biogenesis
GO Biological Process	Response to alcohol
GO Biological Process	Regulation of T cell cytokine production
GO Biological Process	Regulation of response to cytokine stimulus
GO Biological Process	Multicellular organismal homeostasis
GO Biological Process	Response to progesterone
GO Biological Process	Positive regulation of protein localization to nucleus
GO Biological Process	BMP signaling pathway
GO Biological Process	Regulation of chronic inflammatory response
GO Biological Process	Growth hormone receptor signaling pathway via JAK-STAT
GO Biological Process	Negative regulation of MAPK cascade
GO Biological Process	Response to reactive oxygen species
GO Biological Process	Regulation of endothelial cell migration
GO Biological Process	Positive regulation of histone acetylation
GO Biological Process	Regulation of growth
GO Biological Process	Positive regulation of endothelial cell proliferation
GO Biological Process	Positive regulation of immunoglobulin mediated immune response
GO Biological Process	Regulation of transcription by RNA polymerase II
GO Biological Process	Transforming growth factor beta receptor signaling pathway
GO Biological Process	Regulation of hormone levels
GO Biological Process	Platelet activation

GO Biological Process	T cell costimulation
GO Biological Process	Positive regulation of protein import into nucleus
GO Biological Process	Negative regulation of transferase activity
GO Biological Process	Cellular response to interleukin-1
GO Biological Process	Cellular response to tumor necrosis factor
GO Biological Process	Negative regulation of endothelial cell proliferation
GO Biological Process	Positive regulation of glial cell differentiation
GO Biological Process	Regulation of signaling receptor activity
GO Biological Process	Negative regulation of interferon-gamma production
GO Biological Process	Granulocyte migration
GO Biological Process	Positive regulation of secretion by cell
GO Biological Process	T-helper cell lineage commitment
GO Biological Process	Phosphatidylinositol 3-kinase signaling
GO Biological Process	Regulation of interleukin-18 production
GO Biological Process	Positive regulation of smooth muscle cell apoptotic process
GO Biological Process	Interferon-gamma-mediated signaling pathway
GO Biological Process	Negative regulation of T-helper 17 type immune response
GO Biological Process	Negative regulation of alpha-beta T cell activation
GO Biological Process	Mammary gland morphogenesis
GO Biological Process	Regulation of pattern recognition receptor signaling pathway
GO Biological Process	Regulation of glycolytic process
GO Biological Process	Negative regulation of T cell differentiation
GO Biological Process	Regulation of endopeptidase activity
GO Biological Process	Regulation of epithelial cell apoptotic process
GO Biological Process	Negative regulation of lipid localization
GO Biological Process	Protein autophosphorylation
GO Biological Process	Cellular response to interleukin-17
GO Biological Process	Regulation of stress-activated protein kinase signaling cascade
GO Biological Process	Positive regulation of protein-containing complex assembly
GO Biological Process	Positive regulation of peptidyl-serine phosphorylation
GO Biological Process	Organic substance biosynthetic process
GO Biological Process	Regulation of lipid storage
GO Biological Process	Regulation of isotype switching to IgG isotypes
GO Biological Process	B cell receptor signaling pathway
GO Biological Process	Circulatory system development
GO Biological Process	Negative regulation of fat cell differentiation
GO Biological Process	Regulation of cell junction assembly
GO Biological Process	Regulation of platelet activation
GO Biological Process	Positive regulation of heterotypic cell-cell adhesion
GO Biological Process	Protein kinase B signaling
GO Biological Process	Positive regulation of B cell differentiation
GO Biological Process	Negative regulation of activated T cell proliferation
GO Biological Process	Negative regulation of smooth muscle cell proliferation

GO Biological Process	Mononuclear cell migration
GO Biological Process	Positive regulation of neuroinflammatory response
GO Biological Process	Negative regulation of protein kinase activity
GO Biological Process	Cellular response to reactive oxygen species
GO Biological Process	Regulation of cysteine-type endopeptidase activity involved in apoptotic process
GO Biological Process	B cell activation involved in immune response
GO Biological Process	Production of molecular mediator involved in inflammatory response
GO Biological Process	T cell chemotaxis
GO Biological Process	Regulation of interferon-gamma-mediated signaling pathway
GO Biological Process	Positive regulation of T cell mediated immunity
GO Biological Process	Embryonic organ development
GO Biological Process	Negative regulation of adaptive immune response based on somatic recombination of immune receptors built from immunoglobulin superfamily domains
GO Biological Process	Negative regulation of nucleobase-containing compound metabolic process
GO Biological Process	Positive regulation of ERK1 and ERK2 cascade
GO Biological Process	Negative regulation of catalytic activity
GO Biological Process	Astrocyte activation
GO Biological Process	Positive regulation of membrane protein ectodomain proteolysis
GO Biological Process	Response to nutrient levels
GO Biological Process	Regulation of macrophage activation
GO Biological Process	Positive regulation of DNA binding
GO Biological Process	Regulation of mononuclear cell migration
GO Biological Process	Regulation of phosphatidylinositol 3-kinase activity
GO Biological Process	Mammary gland development
GO Biological Process	Peptidyl-tyrosine autophosphorylation
GO Biological Process	Myeloid cell homeostasis
GO Biological Process	Regulation of myeloid leukocyte mediated immunity
GO Biological Process	Negative regulation of tumor necrosis factor production
GO Biological Process	Cellular response to oxidative stress
GO Biological Process	Regulation of intracellular transport
GO Biological Process	Digestive tract development
GO Biological Process	Positive regulation of protein secretion
GO Biological Process	Positive regulation of type I interferon production
GO Biological Process	Lymphocyte homeostasis
GO Biological Process	Negative regulation of biosynthetic process
GO Biological Process	Regulation of fat cell differentiation
GO Biological Process	Regulation of tolerance induction
GO Biological Process	Cellular biosynthetic process
GO Biological Process	Regulation of cellular response to insulin stimulus
GO Biological Process	Negative regulation of epithelial cell proliferation
GO Biological Process	Negative regulation of lipid storage

GO Biological Process	Negative regulation of interleukin-10 production
GO Biological Process	Regulation of hydrolase activity
GO Biological Process	Regulation of muscle cell apoptotic process
GO Biological Process	Mesenchyme development
GO Biological Process	Positive regulation of Wnt signaling pathway
GO Biological Process	Regulation of hormone secretion
GO Biological Process	Response to activity
GO Biological Process	Negative regulation of RNA metabolic process
GO Biological Process	Regulation of reactive oxygen species metabolic process
GO Biological Process	Negative regulation of cellular catabolic process
GO Biological Process	Protein modification process
GO Biological Process	Response to oxidative stress
GO Biological Process	Response to nutrient
GO Biological Process	Positive regulation of alpha-beta T cell activation
GO Biological Process	Positive regulation of peripheral tolerance induction
GO Biological Process	Chemotaxis
GO Biological Process	Intracellular receptor signaling pathway
GO Biological Process	Negative regulation of nervous system development
GO Biological Process	Positive regulation of vitamin D biosynthetic process
GO Biological Process	Positive regulation of cellular response to macrophage colony-stimulating factor stimulus
GO Biological Process	Response to fungus
GO Biological Process	Regulation of toll-like receptor signaling pathway
GO Biological Process	Defense response to virus
GO Biological Process	Negative regulation of tissue remodeling
GO Biological Process	Positive regulation of miRNA-mediated gene silencing
GO Biological Process	Positive regulation of carbohydrate metabolic process
GO Biological Process	Regulation of insulin secretion
GO Biological Process	Positive regulation of interferon-alpha production
GO Biological Process	Regulation of defense response to virus
GO Biological Process	Negative regulation of vascular associated smooth muscle cell proliferation
GO Biological Process	Regulation of ion transport
GO Biological Process	Positive regulation of osteoclast differentiation
GO Biological Process	Negative regulation of alpha-beta T cell differentiation
GO Biological Process	Positive regulation of oligodendrocyte differentiation
GO Biological Process	Negative regulation of lipid catabolic process
GO Biological Process	Response to cholesterol
GO Biological Process	Oligodendrocyte differentiation
GO Biological Process	Positive regulation of intracellular protein transport
GO Biological Process	Negative regulation of inflammatory response to antigenic stimulus
GO Biological Process	Heart development
GO Biological Process	Neutrophil chemotaxis

GO Biological Process	Positive regulation of macrophage activation
GO Biological Process	Regulation of cellular response to stress
GO Biological Process	Positive regulation of amyloid precursor protein catabolic process
GO Biological Process	Chronic inflammatory response to antigenic stimulus
GO Biological Process	Regulation of chronic inflammatory response to antigenic stimulus
GO Biological Process	Negative regulation of macromolecule biosynthetic process
GO Biological Process	Natural killer cell chemotaxis
GO Biological Process	Positive regulation of activated T cell proliferation
GO Biological Process	Regulation of T cell homeostatic proliferation
GO Biological Process	Cellular response to acid chemical
GO Biological Process	Activation of cysteine-type endopeptidase activity involved in apoptotic signaling pathway
GO Biological Process	Positive regulation of plasma cell differentiation
GO Biological Process	Positive regulation of synoviocyte proliferation
GO Biological Process	Positive regulation of extracellular matrix organization
GO Biological Process	Positive regulation of matrix metallopeptidase secretion
GO Biological Process	Regulation of lipid localization
GO Biological Process	Negative regulation of primary miRNA processing
GO Biological Process	Female pregnancy
GO Biological Process	Positive regulation of vascular endothelial growth factor production
GO Biological Process	Calcium-mediated signaling
GO Biological Process	Positive regulation of cellular catabolic process
GO Biological Process	Regulation of system process
GO Biological Process	Negative regulation of transcription, DNA-templated
GO Biological Process	Mesenchymal cell differentiation
GO Biological Process	Epithelial cell proliferation
GO Biological Process	Immunoglobulin production involved in immunoglobulin-mediated immune response
GO Biological Process	Phagocytosis
GO Biological Process	Positive regulation of lipid biosynthetic process
GO Biological Process	Regulation of lipid biosynthetic process
GO Biological Process	Regulation of T-helper 1 type immune response
GO Biological Process	Negative regulation of cell migration
GO Biological Process	Regulation of platelet aggregation
GO Biological Process	Regulation of oxidative stress-induced neuron death
GO Biological Process	Negative regulation of cellular biosynthetic process
GO Biological Process	Regulation of transmembrane transport
GO Biological Process	Histamine secretion by mast cell
GO Biological Process	Regulation of type 2 immune response
GO Biological Process	Sequestering of triglyceride
GO Biological Process	Regulation of protein dephosphorylation
GO Biological Process	Positive regulation of CD4-positive, alpha-beta T cell differentiation
GO Biological Process	Positive regulation of neuron death

GO Biological Process	Post-embryonic development
GO Biological Process	Gene expression
GO Biological Process	Negative regulation of transcription by RNA polymerase II
GO Biological Process	Positive regulation of DNA metabolic process
GO Biological Process	Regulation of stress-activated MAPK cascade
GO Biological Process	Negative regulation of blood vessel endothelial cell migration
GO Biological Process	Positive regulation of phosphatidylinositol 3-kinase activity
GO Biological Process	Defense response to Gram-negative bacterium
GO Biological Process	Negative regulation of extrinsic apoptotic signaling pathway
GO Biological Process	Heterocycle biosynthetic process
GO Biological Process	Regulation of collagen biosynthetic process
GO Biological Process	Regulation of organelle organization
GO Biological Process	Negative regulation of CD4-positive, alpha-beta T cell activation
GO Biological Process	Negative regulation of chronic inflammatory response
GO Biological Process	Positive regulation of neutrophil apoptotic process
GO Biological Process	Positive regulation of histone phosphorylation
GO Biological Process	Tissue remodeling
GO Biological Process	Intestinal epithelial structure maintenance
GO Biological Process	Localization
GO Biological Process	Positive regulation of lamellipodium organization
GO Biological Process	Inflammatory response to antigenic stimulus
GO Biological Process	Negative regulation of angiogenesis
GO Biological Process	Response to hydrogen peroxide
GO Biological Process	Positive regulation of canonical Wnt signaling pathway
GO Biological Process	Activation of protein kinase activity
GO Biological Process	Positive regulation of endothelial cell migration
GO Biological Process	Negative regulation of lipid metabolic process
GO Biological Process	Positive regulation of CD4-positive, CD25-positive, alpha-beta regulatory T cell differentiation
GO Biological Process	Positive regulation of immature T cell proliferation in thymus
GO Biological Process	Positive regulation of Rac protein signal transduction
GO Biological Process	T cell homeostasis
GO Biological Process	Regulation of nitric-oxide synthase activity
GO Biological Process	Cellular response to progesterone stimulus
GO Biological Process	Neutrophil extravasation
GO Biological Process	Negative regulation of CD8-positive, alpha-beta T cell activation
GO Biological Process	Columnar/cuboidal epithelial cell development
GO Biological Process	Negative regulation of response to biotic stimulus
GO Biological Process	Regulation of T-helper cell differentiation
GO Biological Process	Positive regulation of epithelial cell apoptotic process
GO Biological Process	Oligodendrocyte development
GO Biological Process	Regulation of neuron apoptotic process
GO Biological Process	Cellular nitrogen compound biosynthetic process

GO Biological Process	Cellular extravasation
GO Biological Process	Wound healing involved in inflammatory response
GO Biological Process	SMAD protein complex assembly
GO Biological Process	Positive regulation of fever generation
GO Biological Process	Positive regulation of interleukin-23 production
GO Biological Process	Negative regulation of toll-like receptor 2 signaling pathway
GO Biological Process	Tube morphogenesis
GO Biological Process	Regulatory T cell differentiation
GO Biological Process	Positive regulation of cell cycle
GO Biological Process	Regulation of blood vessel remodeling
GO Biological Process	interleukin-27-mediated signaling pathway
GO Biological Process	Regulation of adiponectin secretion
GO Biological Process	Cellular response to nicotine
GO Biological Process	T-helper 17 cell lineage commitment
GO Biological Process	Negative regulation of cardiac muscle hypertrophy in response to stress
GO Biological Process	Positive regulation of vascular associated smooth muscle cell apoptotic process
GO Biological Process	Negative regulation of fibroblast apoptotic process
GO Biological Process	Positive regulation of B cell proliferation
GO Biological Process	Regulation of ion transmembrane transport
GO Biological Process	Regulation of erythrocyte differentiation
GO Biological Process	Regulation of defense response to virus by host
GO Biological Process	Reproduction
GO Biological Process	Epithelium development
GO Biological Process	Positive regulation of organelle organization
GO Biological Process	Negative regulation of osteoblast differentiation
GO Biological Process	Positive regulation of pattern recognition receptor signaling pathway
GO Biological Process	Transcription by RNA polymerase II
GO Biological Process	Vesicle-mediated transport
GO Biological Process	Regulation of chemotaxis
GO Biological Process	Positive regulation of signaling receptor activity
GO Biological Process	System process
GO Biological Process	Respiratory burst involved in defense response
GO Biological Process	Negative regulation of cellular extravasation
GO Biological Process	Positive regulation of antimicrobial humoral response
GO Biological Process	Transcription, DNA-templated
GO Biological Process	Positive regulation of interleukin-18 production
GO Biological Process	Regulation of vascular permeability
GO Biological Process	Regulation of cellular respiration
GO Biological Process	Activated T cell proliferation
GO Biological Process	Mammary gland involution
GO Biological Process	Negative regulation of interferon-gamma-mediated signaling pathway
GO Biological Process	Regulation of hyaluronan biosynthetic process

GO Biological Process	Positive regulation of oxidative stress-induced neuron death
GO Biological Process	Regulation of DNA biosynthetic process
GO Biological Process	Negative regulation of apoptotic signaling pathway
GO Biological Process	Regulation of osteoblast differentiation
GO Biological Process	Regulation of apoptotic signaling pathway
GO Biological Process	Epidermal growth factor receptor signaling pathway
GO Biological Process	Positive regulation of pathway-restricted SMAD protein phosphorylation
GO Biological Process	Positive regulation of protein dephosphorylation
GO Biological Process	Macromolecule metabolic process
GO Biological Process	Positive regulation of stress-activated protein kinase signaling cascade
GO Biological Process	Tissue homeostasis
GO Biological Process	B cell differentiation
GO Biological Process	Fc receptor signaling pathway
GO Biological Process	Regulation of telomerase activity
GO Biological Process	Regulation of endothelial cell apoptotic process
GO Biological Process	Germ cell migration
GO Biological Process	Regulation of alkaline phosphatase activity
GO Biological Process	Endocrine system development
GO Biological Process	Regulation of sequestering of calcium ion
GO Biological Process	Negative regulation of amyloid-beta clearance
GO Biological Process	Organic cyclic compound biosynthetic process
GO Biological Process	Epithelial cell apoptotic process
GO Biological Process	Regulation of cation transmembrane transport
GO Biological Process	Nucleobase-containing compound biosynthetic process
GO Biological Process	B cell proliferation
GO Biological Process	Positive regulation of stress fiber assembly
GO Biological Process	Heart morphogenesis
GO Biological Process	Osteoclast differentiation
GO Biological Process	Positive regulation of platelet activation
GO Biological Process	Negative regulation of collagen biosynthetic process
GO Biological Process	Response to vitamin E
GO Biological Process	Positive regulation of extracellular matrix assembly
GO Biological Process	Positive regulation of chemokine (C-X-C motif) ligand 2 production
GO Biological Process	Tumor necrosis factor-mediated signaling pathway
GO Biological Process	Response to axon injury
GO Biological Process	Regulation of I-kappaB kinase/NF-kappaB signaling
GO Biological Process	Regulation of JNK cascade
GO Biological Process	Biological process involved in symbiotic interaction
GO Biological Process	Regulation of phosphoprotein phosphatase activity
GO Biological Process	Negative regulation of carbohydrate metabolic process
GO Biological Process	Negative regulation of T cell cytokine production
GO Biological Process	Negative regulation of toll-like receptor 4 signaling pathway

GO Biological Process	Negative regulation of neurogenesis
GO Biological Process	Cellular response to lipoteichoic acid
GO Biological Process	Positive regulation of podosome assembly
GO Biological Process	Endothelial cell apoptotic process
GO Biological Process	Negative regulation of T-helper 17 cell differentiation
GO Biological Process	Regulation of metal ion transport
GO Biological Process	Response to purine-containing compound
GO Biological Process	Secretion by cell
GO Biological Process	Sensory organ development
GO Biological Process	Positive regulation of chemotaxis
GO Biological Process	Regulation of calcium ion transport
GO Biological Process	Cellular response to insulin stimulus
GO Biological Process	Mammary gland epithelium development
GO Biological Process	Regulation of protein deacetylation
GO Biological Process	I-kappaB phosphorylation
GO Biological Process	Regulation of epithelial cell differentiation
GO Biological Process	Detection of molecule of bacterial origin
GO Biological Process	Anoikis
GO Biological Process	Positive regulation of platelet aggregation
GO Biological Process	Positive regulation of miRNA maturation
GO Biological Process	Positive regulation of leukocyte migration
GO Biological Process	Blood vessel morphogenesis
GO Biological Process	Response to fatty acid
GO Biological Process	Regulation of cytokine-mediated signaling pathway
GO Biological Process	Negative regulation of neuron apoptotic process
GO Biological Process	Regulation of transmembrane receptor protein serine/threonine kinase signaling pathway
GO Biological Process	Regulation of interleukin-2 production
GO Biological Process	Activation of innate immune response
GO Biological Process	Regulation of T cell tolerance induction
GO Biological Process	Positive regulation of inflammatory response to antigenic stimulus
GO Biological Process	Tyrosine phosphorylation of STAT protein
GO Biological Process	Response to inactivity
GO Biological Process	Positive regulation of Ras protein signal transduction
GO Biological Process	Regulation of insulin receptor signaling pathway
GO Biological Process	Neurotrophin TRK receptor signaling pathway
GO Biological Process	Regulation of morphogenesis of an epithelium
GO Biological Process	Regulation of T-helper 2 cell cytokine production
GO Biological Process	Regulation of cell growth
GO Biological Process	Heart valve development
GO Biological Process	Cellular response to hydrogen peroxide
GO Biological Process	Regulation of cellular carbohydrate metabolic process
GO Biological Process	Negative regulation of transport

GO Biological Process	Lymphocyte mediated immunity
GO Biological Process	Reproductive structure development
GO Biological Process	Negative regulation of leukocyte mediated immunity
GO Biological Process	Negative regulation of type 2 immune response
GO Biological Process	Negative regulation of glycolytic process
GO Biological Process	Negative regulation of telomerase activity
GO Biological Process	Intestinal epithelial cell development
GO Biological Process	Cellular response to retinoic acid
GO Biological Process	Negative regulation of CD4-positive, alpha-beta T cell proliferation
GO Biological Process	Regulation of response to wounding
GO Biological Process	Positive regulation of reactive oxygen species metabolic process
GO Biological Process	Epithelial cell differentiation
GO Biological Process	Developmental process involved in reproduction
GO Biological Process	Positive regulation of immune response to tumor cell
GO Biological Process	sphingosine-1-phosphate receptor signaling pathway
GO Biological Process	Response to leucine
GO Biological Process	Positive regulation of angiogenesis
GO Biological Process	Maintenance of location
GO Biological Process	Pathway-restricted SMAD protein phosphorylation
GO Biological Process	Regulation of neutrophil activation
GO Biological Process	Kidney development
GO Biological Process	Regulation of cartilage development
GO Biological Process	Reproductive process
GO Biological Process	Positive regulation of DNA biosynthetic process
GO Biological Process	Morphogenesis of a branching structure
GO Biological Process	Neutrophil homeostasis
GO Biological Process	Regulation of extracellular matrix disassembly
GO Biological Process	Regulation of organic acid transport
GO Biological Process	Negative regulation of innate immune response
GO Biological Process	Regulation of establishment of endothelial barrier
GO Biological Process	Cranial skeletal system development
GO Biological Process	Response to temperature stimulus
GO Biological Process	Positive regulation of cellular amide metabolic process
GO Biological Process	Embryo development
GO Biological Process	Negative regulation of cytokine-mediated signaling pathway
GO Biological Process	Response to carbohydrate
GO Biological Process	Cellular metabolic process
GO Biological Process	Cellular response to amino acid stimulus
GO Biological Process	Regulation of amino acid import across plasma membrane
GO Biological Process	Regulation of striated muscle tissue development
GO Biological Process	Response to vitamin
GO Biological Process	Regulation of cellular response to growth factor stimulus
GO Biological Process	Epithelial to mesenchymal transition

GO Biological Process	Positive regulation of phosphatidylinositol 3-kinase signaling
GO Biological Process	Dendritic cell chemotaxis
GO Biological Process	Negative regulation of glucose transmembrane transport
GO Biological Process	Negative regulation of interleukin-12 production
GO Biological Process	Regulation of early endosome to late endosome transport
GO Biological Process	Acute inflammatory response
GO Biological Process	Cell-cell signaling
GO Biological Process	Lens development in camera-type eye
GO Biological Process	Positive regulation of cytokine production involved in immune response
GO Biological Process	Chemokine-mediated signaling pathway
GO Biological Process	Regulation of calcium ion transmembrane transport
GO Biological Process	Positive regulation of T-helper 1 type immune response
GO Biological Process	Response to vitamin A
GO Biological Process	Toll-like receptor 4 signaling pathway
GO Biological Process	Positive regulation of lymphocyte apoptotic process
GO Biological Process	Negative regulation of anoikis
GO Biological Process	Regulation of calcium-mediated signaling
GO Biological Process	Regulation of release of sequestered calcium ion into cytosol
GO Biological Process	Epithelial cell development
GO Biological Process	Positive regulation of I-kappaB kinase/NF-kappaB signaling
GO Biological Process	Negative regulation of nervous system process
GO Biological Process	Regulation of cell cycle
GO Biological Process	interleukin-1-mediated signaling pathway
GO Biological Process	Positive regulation of transforming growth factor beta production
GO Biological Process	Detection of other organism
GO Biological Process	Negative regulation of miRNA transcription
GO Biological Process	Regulation of bicellular tight junction assembly
GO Biological Process	Protein metabolic process
GO Biological Process	Negative regulation of cellular component organization
GO Biological Process	Glucose homeostasis
GO Biological Process	Positive regulation of myeloid leukocyte mediated immunity
GO Biological Process	Vascular endothelial growth factor signaling pathway
GO Biological Process	Fc-gamma receptor signaling pathway involved in phagocytosis
GO Biological Process	Positive regulation of glycolytic process
GO Biological Process	Positive regulation of amyloid-beta formation
GO Biological Process	Chemical homeostasis
GO Biological Process	Negative regulation of NF-kappaB transcription factor activity
GO Biological Process	Organic substance metabolic process
GO Biological Process	Regulation of Ras protein signal transduction
GO Biological Process	Regulation of cell projection assembly
GO Biological Process	Positive regulation of cytosolic calcium ion concentration
GO Biological Process	Regulation of protein binding

GO Biological Process	Stimulatory C-type lectin receptor signaling pathway
GO Biological Process	Positive regulation of phosphoprotein phosphatase activity
GO Biological Process	T-helper 1 type immune response
GO Biological Process	Negative regulation of phagocytosis
GO Biological Process	Branching involved in mammary gland duct morphogenesis
GO Biological Process	Positive regulation of cell projection organization
GO Biological Process	Positive regulation of cell division
GO Biological Process	Regulation of anion transport
GO Biological Process	Hyaluronan metabolic process
GO Biological Process	Cellular response to interferon-beta
GO Biological Process	Response to immobilization stress
GO Biological Process	Fc-epsilon receptor signaling pathway
GO Biological Process	Isotype switching
GO Biological Process	Positive regulation of leukocyte adhesion to vascular endothelial cell
GO Biological Process	Hormone secretion
GO Biological Process	Macromolecule biosynthetic process
GO Biological Process	Regulation of synapse organization
GO Biological Process	Metabolic process
GO Biological Process	Positive regulation of JNK cascade
GO Biological Process	Negative regulation of chemokine production
GO Biological Process	Cellular response to platelet-derived growth factor stimulus
GO Biological Process	Positive regulation of nitric-oxide synthase activity
GO Biological Process	Cellular response to angiotensin
GO Biological Process	Response to inorganic substance
GO Biological Process	Anatomical structure formation involved in morphogenesis
GO Biological Process	Neutrophil mediated immunity
GO Biological Process	Nitric oxide metabolic process
GO Biological Process	Negative regulation of receptor signaling pathway via STAT
GO Biological Process	Regulation of vascular endothelial cell proliferation
GO Biological Process	Positive regulation of transcription regulatory region DNA binding
GO Biological Process	Regulation of actin cytoskeleton organization
GO Biological Process	Regulation of cytoskeleton organization
GO Biological Process	Regulation of cell-substrate adhesion
GO Biological Process	Regulation of protein catabolic process
GO Biological Process	Positive regulation of T cell cytokine production
GO Biological Process	Response to muscle stretch
GO Biological Process	Cell adhesion
GO Biological Process	Organophosphate biosynthetic process
GO Biological Process	Negative regulation of small molecule metabolic process
GO Biological Process	Positive regulation of protein localization to membrane
GO Biological Process	Salivary gland morphogenesis
GO Biological Process	Defense response to protozoan
GO Biological Process	Cardiac epithelial to mesenchymal transition

GO Biological Process	Regulation of protein localization to plasma membrane
GO Biological Process	Eye development
GO Biological Process	Thyroid gland development
GO Biological Process	Activin receptor signaling pathway
GO Biological Process	Negative regulation of myoblast differentiation
GO Biological Process	Positive regulation of isotype switching
GO Biological Process	Focal adhesion assembly
GO Biological Process	Response to lipoprotein particle
GO Biological Process	Regulation of anion transmembrane transport
GO Biological Process	Transport
GO Biological Process	Response to toxic substance
GO Biological Process	Heart process
GO Biological Process	Striated muscle contraction
GO Biological Process	Entry into host
GO Biological Process	Positive regulation of lamellipodium assembly
GO Biological Process	Activation of protein kinase B activity
GO Biological Process	Positive regulation of toll-like receptor signaling pathway
GO Biological Process	Lens fiber cell differentiation
GO Biological Process	Positive regulation of plasma membrane bounded cell projection assembly
GO Biological Process	Positive regulation of lipid localization
GO Biological Process	B cell homeostasis
GO Biological Process	Positive regulation of translational initiation
GO Biological Process	Regulation of smooth muscle cell differentiation
GO Biological Process	Positive regulation of protein deacetylation
GO Biological Process	Cardiocyte differentiation
GO Biological Process	Temperature homeostasis
GO Biological Process	Regulation of macrophage derived foam cell differentiation
GO Biological Process	Positive regulation of erythrocyte differentiation
GO Biological Process	Cellular response to lipoprotein particle stimulus
GO Biological Process	Embryonic morphogenesis
GO Biological Process	Negative regulation of type I interferon production
GO Biological Process	Notch signaling pathway
GO Biological Process	Cellular response to extracellular stimulus
GO Biological Process	Endothelial cell proliferation
GO Biological Process	T cell mediated immunity
GO Biological Process	Negative regulation of insulin receptor signaling pathway
GO Biological Process	Regulation of kidney development
GO Biological Process	Regulation of ossification
GO Biological Process	Positive regulation of cell cycle process
GO Biological Process	Response to estradiol
GO Biological Process	Positive regulation of defense response to virus by host
GO Biological Process	Response to fluid shear stress

GO Biological Process	Regulation of cell-matrix adhesion
GO Biological Process	Cellular process involved in reproduction in multicellular organism
GO Biological Process	Negative regulation of macroautophagy
GO Biological Process	Positive regulation of smooth muscle cell migration
GO Biological Process	Positive regulation of interleukin-2 production
GO Biological Process	Extrinsic apoptotic signaling pathway in absence of ligand
GO Biological Process	Regulation of sprouting angiogenesis
GO Biological Process	Positive regulation of morphogenesis of an epithelium
GO Biological Process	Negative regulation of extrinsic apoptotic signaling pathway in absence of ligand
GO Biological Process	Cardiac ventricle development
GO Biological Process	Mesoderm development
GO Biological Process	Negative regulation of transmembrane transport
GO Biological Process	Primary metabolic process
GO Biological Process	Vascular process in circulatory system
GO Biological Process	Regulation of leukocyte chemotaxis
GO Biological Process	Cellular response to hypoxia
GO Biological Process	Positive regulation of fatty acid metabolic process
GO Biological Process	Organonitrogen compound metabolic process
GO Biological Process	Response to organophosphorus
GO Biological Process	Regulation of wound healing
GO Biological Process	Positive regulation of apoptotic signaling pathway
GO Biological Process	Modulation of chemical synaptic transmission
GO Biological Process	Humoral immune response
GO Biological Process	Regulation of macrophage cytokine production
GO Biological Process	Dendritic cell differentiation
GO Biological Process	Rhythmic process
GO Biological Process	Hormone-mediated signaling pathway
GO Cellular Component	Receptor complex
GO Cellular Component	Plasma membrane signaling receptor complex
GO Cellular Component	Cell surface
GO Cellular Component	External side of plasma membrane
GO Cellular Component	Side of membrane
GO Cellular Component	Membrane protein complex
GO Cellular Component	Membrane raft
GO Cellular Component	Phosphatidylinositol 3-kinase complex
GO Cellular Component	Phosphatidylinositol 3-kinase complex, class IA
GO Cellular Component	Cytoplasmic vesicle
GO Cellular Component	Plasma membrane
GO Cellular Component	Integral component of plasma membrane
GO Cellular Component	Transferase complex, transferring phosphorus-containing groups
GO Cellular Component	Extrinsic component of membrane
GO Cellular Component	Protein-containing complex

GO Cellular Component	Phosphatidylinositol 3-kinase complex, class IB
GO Cellular Component	Lipopolysaccharide receptor complex
GO Cellular Component	Transforming growth factor beta ligand-receptor complex
GO Cellular Component	Endosome
GO Cellular Component	Vesicle
GO Cellular Component	Plasma membrane raft
GO Cellular Component	interleukin-12 receptor complex
GO Cellular Component	interleukin-23 receptor complex
GO Cellular Component	Cytosol
GO Cellular Component	Perinuclear region of cytoplasm
GO Cellular Component	Postsynaptic specialization, intracellular component
GO Cellular Component	T cell receptor complex
GO Cellular Component	Endomembrane system
GO Molecular Function	Signaling receptor binding
GO Molecular Function	Cytokine receptor binding
GO Molecular Function	Protein binding
GO Molecular Function	Cytokine activity
GO Molecular Function	Growth factor receptor binding
GO Molecular Function	1-phosphatidylinositol-4-phosphate 3-kinase activity
GO Molecular Function	phosphatidylinositol-4,5-bisphosphate 3-kinase activity
GO Molecular Function	phosphatidylinositol-3,4-bisphosphate 5-kinase activity
GO Molecular Function	1-phosphatidylinositol-3-kinase activity
GO Molecular Function	Phosphotransferase activity, alcohol group as acceptor
GO Molecular Function	Protein kinase activity
GO Molecular Function	Identical protein binding
GO Molecular Function	Kinase activity
GO Molecular Function	Cytokine binding
GO Molecular Function	Binding
GO Molecular Function	Insulin receptor substrate binding
GO Molecular Function	Cytokine receptor activity
GO Molecular Function	Enzyme binding
GO Molecular Function	Non-membrane spanning protein tyrosine kinase activity
GO Molecular Function	Carbohydrate derivative binding
GO Molecular Function	Protein tyrosine kinase activity
GO Molecular Function	Transforming growth factor beta receptor binding
GO Molecular Function	ATP binding
GO Molecular Function	Molecular function regulator activity
GO Molecular Function	Ephrin receptor binding
GO Molecular Function	Transcription factor binding
GO Molecular Function	Phosphoprotein binding
GO Molecular Function	Lipopolysaccharide immune receptor activity
GO Molecular Function	Phosphatase binding
GO Molecular Function	Lipopolysaccharide binding

GO Molecular Function	Transcription coregulator binding
GO Molecular Function	Transcription coactivator binding
GO Molecular Function	Signaling receptor activity
GO Molecular Function	Protein-containing complex binding
GO Molecular Function	RNA polymerase II-specific DNA-binding transcription factor binding
GO Molecular Function	Protein serine kinase activity
GO Molecular Function	Coreceptor activity
GO Molecular Function	Type II transforming growth factor beta receptor binding
GO Molecular Function	Glycosphingolipid binding
GO Molecular Function	Nuclear receptor binding
GO Molecular Function	Promoter-specific chromatin binding

Table S3. KEGG enrichment entry.

category	description	term name
KEGG Pathways	Th17 cell differentiation	hsa04659
KEGG Pathways	Inflammatory bowel disease	hsa05321
KEGG Pathways	Chagas disease	hsa05142
KEGG Pathways	Toxoplasmosis	hsa05145
KEGG Pathways	Hepatitis B	hsa05161
KEGG Pathways	PD-L1 expression and PD-1 checkpoint pathway in cancer	hsa05235
KEGG Pathways	AGE-RAGE signaling pathway in diabetic complications	hsa04933
KEGG Pathways	JAK-STAT signaling pathway	hsa04630
KEGG Pathways	Osteoclast differentiation	hsa04380
KEGG Pathways	Tuberculosis	hsa05152
KEGG Pathways	Yersinia infection	hsa05135
KEGG Pathways	Toll-like receptor signaling pathway	hsa04620
KEGG Pathways	Amoebiasis	hsa05146
KEGG Pathways	Cytokine-cytokine receptor interaction	hsa04060
KEGG Pathways	Kaposi sarcoma-associated herpesvirus infection	hsa05167
KEGG Pathways	T cell receptor signaling pathway	hsa04660
KEGG Pathways	C-type lectin receptor signaling pathway	hsa04625
KEGG Pathways	Leishmaniasis	hsa05140
KEGG Pathways	Pancreatic cancer	hsa05212
KEGG Pathways	Pathways in cancer	hsa05200
KEGG Pathways	Chemokine signaling pathway	hsa04062
KEGG Pathways	Measles	hsa05162
KEGG Pathways	Prolactin signaling pathway	hsa04917
KEGG Pathways	Human T-cell leukemia virus 1 infection	hsa05166
KEGG Pathways	Human cytomegalovirus infection	hsa05163
KEGG Pathways	Influenza A	hsa05164
KEGG Pathways	Proteoglycans in cancer	hsa05205
KEGG Pathways	PI3K-Akt signaling pathway	hsa04151
KEGG Pathways	HIF-1 signaling pathway	hsa04066
KEGG Pathways	Salmonella infection	hsa05132
KEGG Pathways	Chronic myeloid leukemia	hsa05220
KEGG Pathways	EGFR tyrosine kinase inhibitor resistance	hsa01521
KEGG Pathways	Malaria	hsa05144
KEGG Pathways	FoxO signaling pathway	hsa04068
KEGG Pathways	Rheumatoid arthritis	hsa05323
KEGG Pathways	Epstein-Barr virus infection	hsa05169
KEGG Pathways	Legionellosis	hsa05134
KEGG Pathways	Human immunodeficiency virus 1 infection	hsa05170
KEGG Pathways	Cellular senescence	hsa04218

KEGG Pathways	Shigellosis	hsa05131
KEGG Pathways	MicroRNAs in cancer	hsa05206
KEGG Pathways	Growth hormone synthesis, secretion and action	hsa04935
KEGG Pathways	Type II diabetes mellitus	hsa04930
KEGG Pathways	Colorectal cancer	hsa05210
KEGG Pathways	Th1 and Th2 cell differentiation	hsa04658
KEGG Pathways	Herpes simplex virus 1 infection	hsa05168
KEGG Pathways	Viral protein interaction with cytokine and cytokine receptor	hsa04061
KEGG Pathways	Hepatitis C	hsa05160
KEGG Pathways	Insulin resistance	hsa04931
KEGG Pathways	Fc epsilon RI signaling pathway	hsa04664
KEGG Pathways	TNF signaling pathway	hsa04668
KEGG Pathways	Acute myeloid leukemia	hsa05221
KEGG Pathways	Viral carcinogenesis	hsa05203
KEGG Pathways	Platelet activation	hsa04611
KEGG Pathways	Pertussis	hsa05133
KEGG Pathways	Relaxin signaling pathway	hsa04926
KEGG Pathways	Fluid shear stress and atherosclerosis	hsa05418
KEGG Pathways	B cell receptor signaling pathway	hsa04662
KEGG Pathways	Non-alcoholic fatty liver disease	hsa04932
KEGG Pathways	Gastric cancer	hsa05226
KEGG Pathways	IL-17 signaling pathway	hsa04657
KEGG Pathways	Hepatocellular carcinoma	hsa05225
KEGG Pathways	Pathogenic Escherichia coli infection	hsa05130
KEGG Pathways	Thyroid hormone signaling pathway	hsa04919
KEGG Pathways	African trypanosomiasis	hsa05143
KEGG Pathways	Graft-versus-host disease	hsa05332
KEGG Pathways	Type I diabetes mellitus	hsa04940
KEGG Pathways	ErbB signaling pathway	hsa04012
KEGG Pathways	Signaling pathways regulating pluripotency of stem cells	hsa04550
KEGG Pathways	Necroptosis	hsa04217
KEGG Pathways	mTOR signaling pathway	hsa04150
KEGG Pathways	Longevity regulating pathway	hsa04211
KEGG Pathways	Hematopoietic cell lineage	hsa04640
KEGG Pathways	TGF-beta signaling pathway	hsa04350
KEGG Pathways	Endocrine resistance	hsa01522
KEGG Pathways	Prostate cancer	hsa05215
KEGG Pathways	Alzheimer disease	hsa05010
KEGG Pathways	NF-kappa B signaling pathway	hsa04064
KEGG Pathways	NOD-like receptor signaling pathway	hsa04621
KEGG Pathways	Cholinergic synapse	hsa04725

KEGG Pathways	VEGF signaling pathway	hsa04370
KEGG Pathways	Sphingolipid signaling pathway	hsa04071
KEGG Pathways	Natural killer cell mediated cytotoxicity	hsa04650
KEGG Pathways	Renal cell carcinoma	hsa05211
KEGG Pathways	Adipocytokine signaling pathway	hsa04920
KEGG Pathways	Non-small cell lung cancer	hsa05223
KEGG Pathways	Central carbon metabolism in cancer	hsa05230
KEGG Pathways	Autophagy - animal	hsa04140
KEGG Pathways	Apoptosis	hsa04210
KEGG Pathways	Insulin signaling pathway	hsa04910
KEGG Pathways	Glioma	hsa05214
KEGG Pathways	Human papillomavirus infection	hsa05165
KEGG Pathways	Antifolate resistance	hsa01523
KEGG Pathways	Phospholipase D signaling pathway	hsa04072
KEGG Pathways	Allograft rejection	hsa05330
KEGG Pathways	Aldosterone-regulated sodium reabsorption	hsa04960
KEGG Pathways	Fc gamma R-mediated phagocytosis	hsa04666
KEGG Pathways	Inflammatory mediator regulation of TRP channels	hsa04750
KEGG Pathways	Prion disease	hsa05020
KEGG Pathways	Choline metabolism in cancer	hsa05231
KEGG Pathways	Intestinal immune network for IgA production	hsa04672
KEGG Pathways	Neurotrophin signaling pathway	hsa04722
KEGG Pathways	AMPK signaling pathway	hsa04152
KEGG Pathways	Endometrial cancer	hsa05213
KEGG Pathways	Longevity regulating pathway - multiple species	hsa04213
KEGG Pathways	Apelin signaling pathway	hsa04371
KEGG Pathways	Estrogen signaling pathway	hsa04915
KEGG Pathways	GnRH secretion	hsa04929
KEGG Pathways	Antigen processing and presentation	hsa04612
KEGG Pathways	Cell adhesion molecules	hsa04514
KEGG Pathways	Adherens junction	hsa04520
KEGG Pathways	Platinum drug resistance	hsa01524
KEGG Pathways	Bacterial invasion of epithelial cells	hsa05100
KEGG Pathways	Breast cancer	hsa05224
KEGG Pathways	Inositol phosphate metabolism	hsa00562
KEGG Pathways	Melanoma	hsa05218
KEGG Pathways	MAPK signaling pathway	hsa04010
KEGG Pathways	Axon guidance	hsa04360
KEGG Pathways	Small cell lung cancer	hsa05222
KEGG Pathways	Progesterone-mediated oocyte maturation	hsa04914
KEGG Pathways	Focal adhesion	hsa04510
KEGG Pathways	Primary immunodeficiency	hsa05340

KEGG Pathways	Rap1 signaling pathway	hsa04015
KEGG Pathways	cAMP signaling pathway	hsa04024
KEGG Pathways	Regulation of actin cytoskeleton	hsa04810
KEGG Pathways	Carbohydrate digestion and absorption	hsa04973
KEGG Pathways	Ras signaling pathway	hsa04014
KEGG Pathways	Autoimmune thyroid disease	hsa05320
KEGG Pathways	Endocytosis	hsa04144
KEGG Pathways	Regulation of lipolysis in adipocytes	hsa04923
KEGG Pathways	Spinocerebellar ataxia	hsa05017
KEGG Pathways	Cytosolic DNA-sensing pathway	hsa04623
KEGG Pathways	Epithelial cell signaling in Helicobacter pylori infection	hsa05120
KEGG Pathways	Hypertrophic cardiomyopathy	hsa05410
KEGG Pathways	Phosphatidylinositol signaling system	hsa04070
KEGG Pathways	Systemic lupus erythematosus	hsa05322
KEGG Pathways	Asthma	hsa05310
KEGG Pathways	Leukocyte transendothelial migration	hsa04670
KEGG Pathways	Thyroid cancer	hsa05216
KEGG Pathways	Bladder cancer	hsa05219
KEGG Pathways	Oxytocin signaling pathway	hsa04921
KEGG Pathways	Hippo signaling pathway	hsa04390
KEGG Pathways	Transcriptional misregulation in cancer	hsa05202
KEGG Pathways	Long-term depression	hsa04730
KEGG Pathways	RIG-I-like receptor signaling pathway	hsa04622
KEGG Pathways	Gap junction	hsa04540
KEGG Pathways	GnRH signaling pathway	hsa04912

Table S4. Binding Energies and RMSD of the 26 Compounds in the Active Pocket Site of the Catalytic Domain of topoisomerase IV *S. pneumoniae* (PDB: 4KVB).

Compound	S kcal/mole	RMSD_Refine
Sinapinic acid	-6.45606	1.0153158
Mannitol	-5.17358	2.2796125
Trehalose	-6.15	1.9633542
Vanillic Acid	-4.80908	1.5984399
Fluocinolone	-5.64679	3.0373192
Azelaic acid	-5.23167	1.4997731
p-Coumaric acid	-4.85833	1.8931016
Diflunisal	-5.64061	1.5190358
Ursinoic acid	-5.8381	3.0383058
Kaempferol	-5.8422	2.1282849
Fluorohydroxyandrostenedione	-5.43049	2.3739686
Dihydroactinidiolide	-4.9523	1.8233382
Ferulic acid	-5.60912	1.7119412
Genistein	-5.4206	1.5704401
Dibutyl Phthalate	-6.96462	1.7006348
Arachidonic acid	-6.18087	1.502454
Oleic Acid	-6.69097	1.5575464
11,13-Dimethyl-12-tetradecen-1-ol acetate	-6.27319	1.5021387
Crocetin	-5.11243	1.7961679
Gamma-Linolenic Acid (GLA)	-6.6353	1.6371549
10,13-Octadecadienoic acid	-6.57336	1.87963
1,2-Cyclohexanedicarboxylic acid, cyclohexyl isohexyl ester	-6.83671	0.70289886
Rhamnose	-4.24025	1.6688143
Palmitic acid	-6.92266	1.9477121
Caffeic acid	-5.33242	1.3514175
Dinonyl cyclohexane-1,2-dicarboxylate	-7.53856	1.901482
Levofloxacin	-5.59781	1.943016
